# Refining Prognosis and Treatment Strategies Beyond the Barcelona Clinic Liver Cancer Stage in Hepatocellular Carcinoma with Lung Metastases: A Multicenter Cohort Study

**DOI:** 10.1002/mco2.70306

**Published:** 2025-08-16

**Authors:** Feng Xia, Qian Chen, Chenyang Li, Huifang Liang, Qiao Zhang, Zhiyuan Huang, Zhenheng Wu, Huaxuan Yin, Liping Liu, Jun Zheng, Hengyi Gao, Guobing Xia, Li Ren, Wanguang Zhang, Xiaoping Chen, Jing Yan, Bixiang Zhang, Huilan Zhang, Zhao Huang

**Affiliations:** ^1^ Department of Hepatic Surgery Tongji Hospital Tongji Medical College of Huazhong University of Science and Technology Wuhan Hubei China; ^2^ Department of Hepatobiliary Surgery First Affiliated Hospital of Shihezi University Shihezi Xinjiang China; ^3^ Department of Hepatic Surgery Zhongshan People's Hospital Affiliated to Guangdong Medical University Zhongshan Guangdong China; ^4^ Department of General Surgery General Hospital of Central Theater Command Wuhan Hubei China; ^5^ Department of Hepatopancreatobiliary Surgery The First Affiliated Hospital of Fujian Medical University Fuzhou China; ^6^ Department of Hepatic‐Biliary‐Pancreatic Surgery The First People's Hospital of Foshan Foshan China; ^7^ Department of Hepatobiliary Surgery Shenzhen People's Hospital Shenzhen Guangdong China; ^8^ Department of Science and Education Shenzhen Baoan District People's Hospital Shenzhen Guangdong China; ^9^ Department of Hepatobiliary and Pancreatic Surgery Shenzhen Longhua District People's Hospital Shenzhen Guangdong China; ^10^ Department of Hepatobiliary and Pancreatic Surgery Huangshi Central Hospital, Hubei Polytechnic University Huangshi Hubei China; ^11^ Department of Hepatobiliary Surgery Affiliated Hospital of Qinghai University Xining Qinghai China; ^12^ Department of Ultrasound in Medicine The Second Affiliated Hospital of Zhejiang University School of Medicine Hangzhou Zhejiang China; ^13^ Department of Respiratory and Critical Care Medicine National Health Commission Key Laboratory of Respiratory Diseases, Tongji Hospital, Tongji Medical College, Huazhong University of Science and Technology Wuhan China

**Keywords:** hepatocellular carcinoma, hepatectomy, immune checkpoint inhibitors, lung metastasis, prognostic factors, radiofrequency ablation

## Abstract

Lung metastasis is the most common site of extrahepatic spread in hepatocellular carcinoma (HCC) and is associated with significantly poorer outcomes. Current guidelines classify these patients as Barcelona Clinic Liver Cancer (BCLC) stage C, recommending systemic therapy alone. However, this one‐size‐fits‐all approach may overlook potential benefits in selected patients. In this multicenter cohort study of 1203 HCC patients—including 119 with lung metastases—we evaluated prognostic factors and treatment outcomes. Lung metastasis significantly reduced overall survival, both before and after propensity score matching. However, among patients with early‐stage intrahepatic tumors, curative locoregional treatments such as hepatectomy or radiofrequency ablation improved survival and led to outcomes comparable to those without metastasis. Systemic therapies including tyrosine kinase inhibitors (TKIs) and immune checkpoint inhibitors (ICIs) prolonged survival, and combination regimens yielding the greatest benefit. Interestingly, lung metastases impaired intrahepatic response to systemic monotherapy, but this effect was mitigated by combining TKIs with ICIs. These findings suggest that a subset of HCC patients with lung metastases may benefit from individualized, multimodal treatment strategies, challenging the current staging framework and supporting a more refined, personalized therapeutic approach in this population.

## Graphical abstract

This multicenter cohort study revealed that lung metastasis worsens prognosis in hepatocellular carcinoma. However, selected patients with early‐stage intrahepatic tumors benefit from hepatectomy or RFA, achieving outcomes comparable to nonmetastatic cases. Combination therapy with TKIs and ICIs further improves survival. These findings support personalized treatment beyond the current BCLC stage C classification.

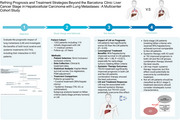

## 1 Introduction

Hepatocellular carcinoma (HCC) remains one of the most lethal malignancies worldwide, accounting for over 800,000 deaths annually and ranking as the second leading cause of cancer‐related mortality in China. The high mortality is largely attributed to its aggressive biological behavior—including vascular invasion, poor differentiation, and early metastasis—and limited responsiveness in advanced stages. With recent advances in systemic therapy, particularly the use of immune checkpoint inhibitors (ICIs) and tyrosine kinase inhibitors (TKIs), treatment paradigms for HCC have shifted toward multimodal strategies that combine systemic and locoregional approaches, such as resection or ablation [1, 2]. Distant metastases represent a major challenge in HCC management, and the lung is the most common extrahepatic site, involved in approximately 30–50% of metastatic cases. Notably, lung metastases are present at initial diagnosis in about 6% of HCC patients [3, 4, 5, 6]. However, there is still no consensus on optimal treatment strategies for this population. Most current guidelines assign these patients to Barcelona Clinic Liver Cancer (BCLC) stage C and recommend systemic therapy alone, often resulting in conservative and uniform management approaches [7]. Despite this, increasing evidence suggests that outcomes may vary significantly depending on intrahepatic tumor burden, metastatic profile, and therapeutic interventions. Yet, data on the prognostic impact of lung metastasis (LM), the efficacy of systemic and locoregional treatments, and the potential benefit of combination therapy in this population remain limited. Therefore, there is a critical need to better define prognosis and identify effective, personalized treatment strategies for HCC patients with LM.

Previous studies have consistently demonstrated that the presence of lung metastases in HCC patients is associated with significantly worse prognosis [8, 9]. Based on large population‐based datasets such as Surveillance, Epidemiology, and End Results (SEER) and institutional cohorts, LM has been identified as a strong negative prognostic factor. Given its relatively high frequency, more effective and individualized treatment strategies for HCC with LM (HCCLM) patients are urgently needed. According to the latest BCLC staging system, all patients with LM are categorized as stage C and are primarily recommended for systemic therapy—typically involving ICIs or TKIs. However, whether all LM patients should receive uniform treatment remains debatable. Studies from East Asia have reported that resection of isolated lung lesions can improve survival in carefully selected patients [10, 11]. Nevertheless, such opportunities are rare, as most LM cases involve multiple lesions at diagnosis, limiting the feasibility of pulmonary metastasectomy. In light of this, the potential benefit of curative treatment directed at the primary hepatic tumor—such as hepatectomy or radiofrequency ablation (RFA)—has garnered increasing interest. Meanwhile, systemic therapies have become the cornerstone of HCCLM management, but their effectiveness may vary based on tumor burden and metastatic patterns. Notably, patients with LM may respond differently to ICI or TKI monotherapy. These considerations raise a key question: can certain subgroups, particularly those with favorable intrahepatic tumor characteristics, benefit from more aggressive or combined therapeutic approaches?

To address these gaps, we conducted a large, multicenter cohort study to evaluate the prognostic impact of LM in HCC and explore individualized treatment strategies. By comparing patients with and without lung metastases and analyzing the effects of systemic therapies (TKIs, ICIs, and their combination) and curative locoregional interventions, we aimed to identify clinical subsets of HCCLM patients who may benefit from more aggressive, multimodal treatment. Ultimately, our goal was to move beyond the current uniform classification of all LM patients as BCLC stage C, and to propose a refined, stratified management approach that improves survival outcomes in this challenging population.

## 2 Results

### 2.1 Baseline Comparison of HCC Patients With and Without Pulmonary Metastasis Before and After PSM

**FIGURE 1:**
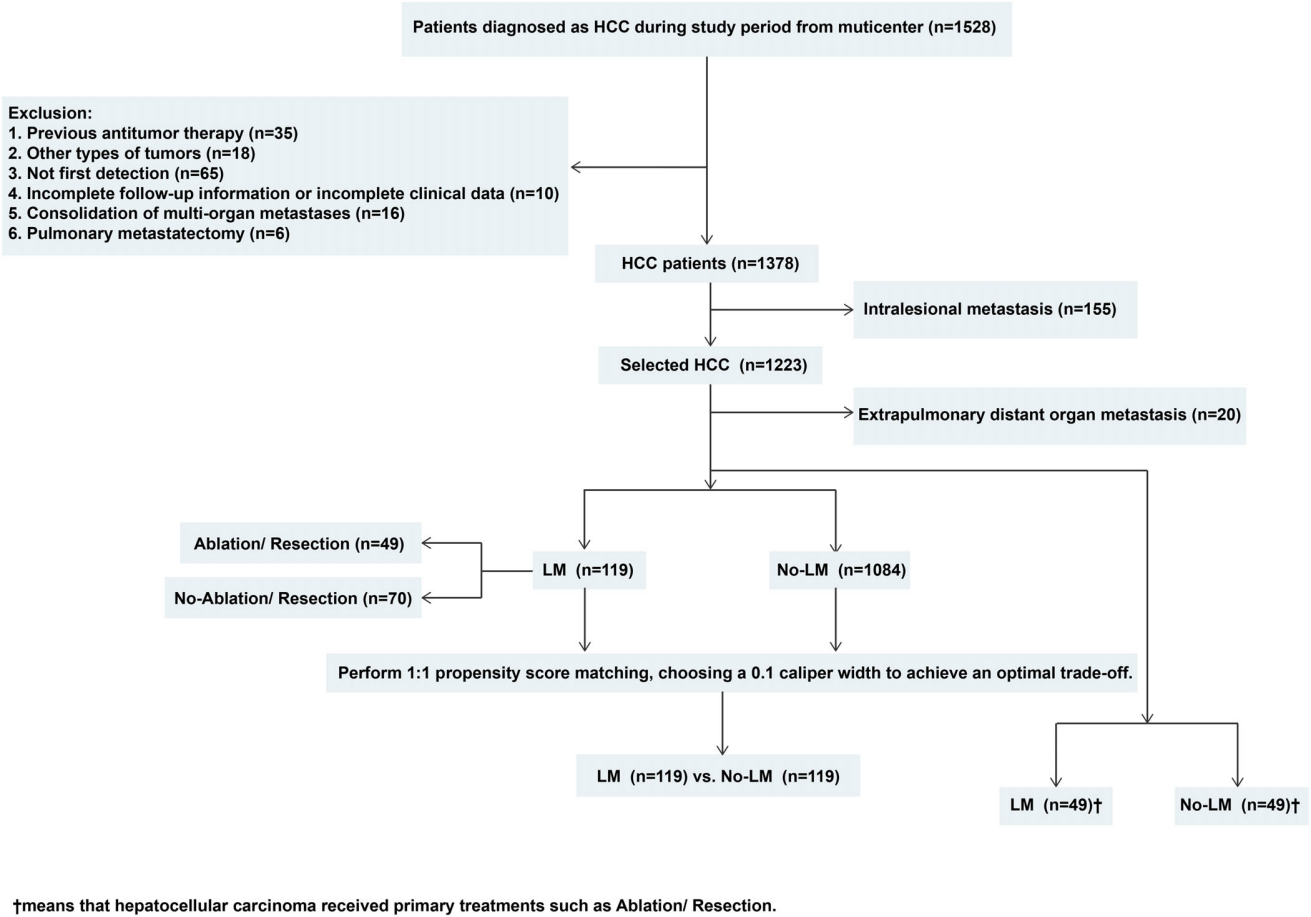
Flow chart for inclusion and exclusion of hepatocellular carcinoma (HCC) patients.

A retrospective consecutive collection was conducted on 1203 patients with HCC treated at eleven centers in China. Among them, there were 119 patients with initial LM. The flowchart of strict inclusion and exclusion criteria for all patients is presented in Figure 1. A comparison of baseline comorbidities between included and excluded patients revealed no significant differences in all variable, suggesting minimal selection bias related to these variables (Table S1). The number of patients from each center is provided in Table S2. Among patients with LM, preoperative imaging showed larger tumor diameters, with 64 patients (53.8%) having tumors larger than 5 cm. Additionally, a higher proportion of patients had portal vein tumor thrombus (PVTT), with 26 patients (21.8%) affected. Serum tumor marker alpha‐fetoprotein (AFP) levels upon admission were also higher, with 86 patients (72.3%) having levels exceeding 400 ng/ml.

Furthermore, among HCCLM patients, 23 (19.3%) underwent primary liver tumor resection, and 27 (22.7%) received primary RFA treatment. The proportions of patients using TKI and ICI were comparable between the two groups. In the LM group, 25 patients, accounting for 21.0%, received combined treatment with both drugs. After 1:1 propensity score matching (PSM), the distribution of variables between the LM group and no‐LM group was balanced, with all *p* values exceeding 0.05. Standardized mean differences (SMD) for all baseline covariates before and after PSM were calculated, and all postmatching SMD values were <0.1, indicating adequate balance between the matched groups (Table S3). Additionally, common comorbidities including diabetes mellitus, hypertension, cardiovascular disease, chronic kidney disease, and chronic obstructive pulmonary disease (COPD) were evaluated. These comorbidities showed no significant difference between LM and no‐LM groups before or after PSM (Table 1). The majority of patients with pulmonary metastasis had multiple metastatic lesions (75.6%), and most of the largest nodule is smaller than 2 cm (80.7%). Remaining details refer to Table S4.

**TABLE 1:** Baseline characteristics of hepatocellular carcinoma patients with/without lung metastasis (LM) in eleven centers before PSM and after PSM (*n* = 1203).

	Before‐PSM			After‐PSM		
	No‐LM (*n* = 1084)	LM (*n* = 119)	*p* Value^a^	No‐LM (*n* = 119)	LM (*n* = 119)	*p* Value^a^
Gender (%)			0.148			0.826
Male	932 (86.0)	108 (90.8)		107 (89.9)	108 (90.8)	
Female	152 (14.0)	11 (9.2)		12 (10.1)	11 (9.2)	
Age (%)			0.034			0.515
<60 y	368 (33.9)	52 (43.7)		57 (47.9)	52 (43.7)	
≥60 y	716 (66.1)	67 (56.3)		62 (52.1)	67 (56.3)	
Tumor max length (%)			<0.001			0.517
<5 cm	814 (75.1)	55 (46.2)		60 (50.4)	55 (46.2)	
≥5 cm	270 (24.9)	64 (53.8)		59 (49.6)	64 (53.8)	
Tumor number (%)			0.733			0.389
Single	817 (75.4)	88 (73.9)		82 (68.9)	88 (73.9)	
Multiple	267 (24.6)	31 (26.1)		37 (31.1)	31 (26.1)	
AFP (%)			<0.001			0.774
<400 ng/mL	563 (51.9)	33 (27.7)		35 (29.4)	33 (27.7)	
≥400 ng/mL	521 (48.1)	86 (72.3)		84 (70.6)	86 (72.3)	
CSPH (%)			<0.001			0.794
No	780 (72.0)	67 (56.3)		65 (54.6)	67 (56.3)	
Yes	304 (28.0)	52 (43.7)		54 (45.4)	52 (43.7)	
PVTT (%)			<0.001			0.645
No	973 (91.4)	93 (78.2)		90 (75.6)	93 (78.2)	
Yes	91 (8.6)	26 (21.8)		29 (24.4)	26 (21.8)	
HBsAg (%)			0.885			0.851
No	151 (13.9)	16 (13.4)		17 (14.3)	16 (13.4)	
Yes	933 (86.1)	103 (86.6)		102 (85.7)	103 (86.6)	
ALBI grade (%)			0.268			0.941
1	108 (10.0)	16 (13.4)		15 (12.6)	16 (13.4)	
2	802 (74.0)	80 (67.2)		79 (66.4)	80 (67.2)	
3	174 (16.1)	23 (19.3)		25 (21.0)	23 (19.3)	
ALB (%)			0.252			0.683
<35 g/L	336 (31.0)	43 (36.1)		40 (33.6)	43 (36.1)	
≥35 g/L	748 (69.0)	76 (63.9)		79 (66.4)	76 (63.9)	
ALT (%)			0.221			0.475
<100 U/L	846 (78.0)	87 (73.1)		82 (68.9)	87 (73.1)	
≥100 U/L	238 (22.0)	32 (26.9)		37 (31.1)	32 (26.9)	
AST (%)			0.081			0.794
<80 U/L	574 (53.0)	53 (44.5)		51 (42.9)	53 (44.5)	
≥80 U/L	510 (47.0)	66 (55.5)		68 (57.1)	66 (55.5)	
ALP (%)			0.573			0.645
<100 U/L	822 (75.8)	93 (78.2)		90 (75.6)	93 (78.2)	
≥100 U/L	262 (24.2)	26 (21.8)		29 (24.4)	26 (21.8)	
GGT (%)			0.280			0.695
<60 U/L	563 (51.9)	68 (57.1)		65 (54.6)	68 (57.1)	
≥60 U/L	521 (48.1)	51 (42.9)		54 (45.4)	51 (42.9)	
Smoking history (%)			<0.001			0.873
No	378 (34.9)	27 (22.7)		24 (20.2)	27 (22.7)	
≤5 y	537 (49.5)	46 (38.7)		49 (41.2)	46 (38.7)	
>5 y	169 (15.6)	46 (38.7)		46 (38.7)	46 (38.7)	
Drinking history (%)			0.245			0.770
No	835 (77.0)	86 (72.3)		88 (73.9)	86 (72.3)	
Yes	249 (23.0)	33 (27.7)		31 (26.1)	33 (27.7)	
RFA of the primary site (%)			<0.001			0.453
No	618 (57.0)	92 (77.3)		87 (73.1)	92 (77.3)	
Yes	466 (43.0)	27 (22.7)		32 (26.9)	27 (22.7)	
Surgery of the primary site (%)			<0.001			0.631
No	672 (62.0)	97 (81.5)		93 (78.2)	97 (81.5)	
Yes	412 (38.0)	22 (18.5)		26 (21.8)	22 (18.5)	
Antiviral therapy (%)			0.912			0.685
No	379 (35.0)	41 (34.5)		44 (37.0)	41 (34.5)	
Yes	705 (65.0)	78 (65.5)		75 (63.0)	78 (65.5)	
Drug usage (%)			0.001			0.753
No use	281 (25.9)	15 (12.6)		18 (15.1)	15 (12.6)	
Only TKI	292 (26.9)	40 (33.6)		43 (36.1)	40 (33.6)	
Only ICI	227 (20.9)	39 (32.8)		39 (32.8)	39 (32.8)	
TKI+ICI	284 (26.2)	25 (21.0)		19 (16.0)	25 (21.0)	
Time to recurrence (months [IQR])						
RFA	11.0 (5.0–25.0)	/		12.0 (6.0–24.0)	/	
Surgery	18.0 (6.0–29.0)	/		19.0 (7.0–30.0)	/	
Comorbidities						
Diabetes mellitus (%)	152 (14.0)	18 (15.1)	0.721	17 (14.3)	18 (15.1)	0.832
Hypertension (%)	203 (18.7)	25 (21.0)	0.524	24 (20.2)	25 (21.0)	0.854
Cardiovascular disease (%)	92 (8.5)	12 (10.1)	0.493	11 (9.2)	12 (10.1)	0.815
Chronic kidney disease (%)	48 (4.4)	5 (4.2)	0.926	5 (4.2)	5 (4.2)	1.000
COPD (%)	28 (2.6)	4 (3.4)	0.591	3 (2.5)	4 (3.4)	0.701

The values in parentheses are percentages unless indicated otherwise.

*Abbreviations*: COPD: chronic obstructive pulmonary disease; PSM: propensity score matching; LM: lung metastasis; PVTT: portal vein tumor thrombosis; AFP: alpha‐fetoprotein; HCC: hepatocellular carcinoma; HBsAg: hepatitis B surface antigen; ALBI: albumin–bilirubin grade; ALT: alanine aminotransferase; AST: aspartate aminotransferase; ALP: alkaline phosphatase; GGT: γ‐glutamyl transpeptidase; RFA: radiofrequency ablation; TKI: tyrosine kinase inhibitor; ICI: immune checkpoint inhibitor.

^a^ *χ* ^2^ test with Yates’ correction.

### 2.2 Prognosis Comparison Between LM and no‐LM Groups Before and After PSM

**FIGURE 2:**
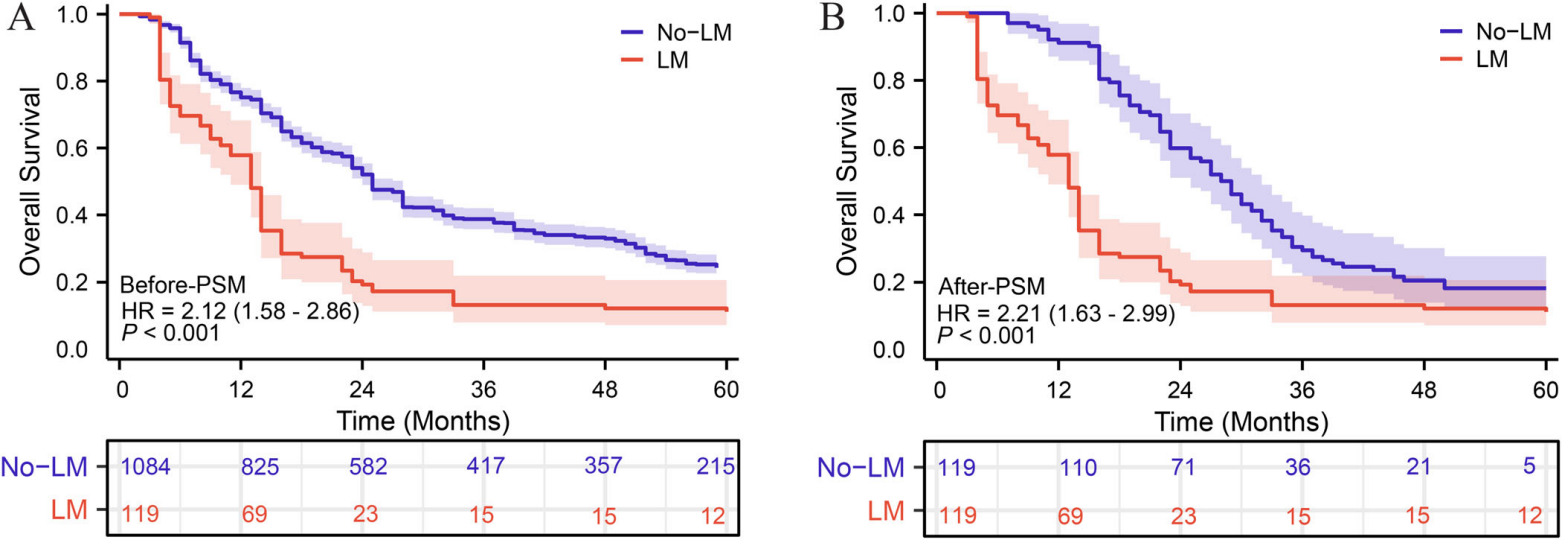
Overall survival rates between patients with lung metastasis (LM) and those without lung metastasis (no‐LM) in all HCC patients. (A) Overall survival (OS) before propensity score matching. (B) OS after propensity score matching.

Before PSM, the 1, 3, and 5‐year overall survival rates in the no‐LM group were 91.6, 56.4, and 30.9%, respectively, while in the LM group, the rates were 83.8, 30.5, and 9.5%, respectively. Kaplan–Meier curves showed a significant statistical difference in survival rates between the two groups [LM vs. no‐LM—hazard ratio [HR]: 2.122 (95% confidence interval [CI]: 1.581–2.862; *p* < 0.001)]. After 1:1 PSM, the 1, 3, and 5‐year overall survival rates in the no‐LM group were 89.7, 55.6, and 21.3%, respectively. There was a statistically significant difference in overall survival rates between the two groups (*p* < 0.05) [LM vs. no‐LM—HR: 2.213 (95% CI: 1.634–2.990; *p* < 0.001)] (Figure 2).

The multivariate model included variables demonstrating a *p* value below 0.05 in univariate analysis. Multivariate logistic regression analysis showed that tumor max length (OR [odds ratio] = 1.455 [1.108–1.826], *p* = 0.018), tumor number (OR = 1.500 [1.187–1.942], *p* < 0.001), AFP (OR = 1.322 [1.082–1.916], *p* < 0.001), PVTT (OR = 1.788 [1.365–2.282], *p* < 0.001), and smoking history (OR = 2.089 [1.496–3.281], *p* < 0.001) were significant factors (Table S5).

### 2.3 Univariate and Multivariate Cox Regression Models Identifying Prognostic Factors in Whole HCC Patients and HCCLM Patients

All variance inflation factor values for variables included in the multivariate Cox model were <2.0, indicating no significant multicollinearity (Table S6). Significant variables from the univariate Cox regression analysis (*p* < 0.05) were included in the multivariate Cox model. The results showed that LM (HR = 1.497 [1.170–2.113], *p* < 0.001), RFA of the primary site (HR = 1.550 [1.153–2.165], *p* < 0.001), surgery of the primary site (HR = 1.719 [1.288–2.630], *p* < 0.001), tumor max length (HR = 1.564 [1.166–2.138], *p* = 0.022), tumor number (HR = 1.610 [1.254–2.230], *p* < 0.001), AFP (HR = 1.581 [1.379–2.170], *p* = 0.018), clinically significant portal hypertension (CSPH) (HR = 1.691 [1.210–2.277], *p* = 0.037), PVTT (HR = 2.087 [1.726–2.612], *p* < 0.001), and drug usage's TKI+ICI (HR = 1.628 [1.316–2.267], *p* < 0.001) were significant predictors (Table 2). Common comorbidities did not emerge as independent prognostic factors for overall survival in the multivariate Cox analysis. In the multivariate Cox analysis conducted specifically in HCC patients with LM (Table S7), both ICI and TKI were independently associated with improved overall survival (HR = 1.341–1.564). This finding was further supported by Kaplan–Meier survival analysis (Figure S1), which showed that patients receiving ICI and/or TKI had significantly better survival compared with those who received neither treatment (*p* < 0.001). Additionally, LM characteristics—including the number and size of metastatic nodules, as well as progression status—were also significant prognostic factors, with multiple or larger nodules and progressive disease associated with worse survival outcomes.

**TABLE 2:** Univariate and multivariate Cox regression analyses of prognosis factors associated with hepatocellular carcinoma.

			Univariate analysis			Multivariate analysis		
Variables	Number	%	*p*	HR	95% CI	*p*	HR	95% CI
Gender			0.277					
Male	1040	86.5%		1.112	0.905–1.346			
Female	163	13.5%		Ref	–			
Age (years)			0.385					
<60	420	35.0%		Ref	–			
≥60	783	65.0%		1.511	0.764–1.921			
Tumor max length (cm)			0.033			0.022		
<5	869	72.2%		Ref	–		Ref	–
≥5	334	27.8%		1.689	1.255–2.372		1.564	1.166–2.138
Tumor number			<0.001			<0.001		
Single	905	75.2%		Ref	–		Ref	–
Multiple	298	24.8%		1.822	1.513–2.481		1.610	1.254–2.230
AFP (ng/mL)			<0.001			0.018		
<400	596	49.5%		Ref	–		Ref	–
≥400	607	50.5%		1.402	1.096–1.832		1.581	1.379–2.170
CSPH			0.016			0.037		
No	847	70.4%		Ref	–		Ref	–
Yes	356	29.6%		1.720	1.313–2.459		1.691	1.210–2.277
PVTT			0.001			<0.001		
No	1066	88.6%		Ref	–		Ref	–
Yes	117	11.4%		2.820	2.411–3.412		2.087	1.726–2.612
ALBI			0.127					
1	124	10.3%		Ref	–			
2–3	1079	89.7%		1.205	0.790–1.812			
ALB (g/L)			0.523					
<35	379	31.5%		Ref	–			
≥35	824	68.5%		0.911	0.690–1.288			
ALT (U/L)			0.346					
<100	933	77.6%		Ref	–			
≥100	270	22.4%		1.478	0.746–1.622			
AST (U/L)			0.422					
<80	627	52.1%		Ref	–			
≥80	576	47.9%		1.293	0.850–1.792			
ALP (U/L)			0.338					
<100	915	76.1%		Ref	–			
≥100	288	23.9%		1.122	0.683–1.691			
GGT (U/L)			<0.001			0.221		
<60	631	52.5%		Ref	–		Ref	–
≥60	572	47.5%		1.199	1.078–1.861		1.088	0.876‐1.326
Drinking history			0.288					
No	921	76.6%		Ref	–			
Yes	282	23.4%		1.312	0.793–1.920			
Smoking history			0.319					
No	405	33.7%		Ref	–			
Yes	798	66.3%		1.351	0.698–1.922			
RFA of the primary site			<0.001			<0.001		
No	710	59.0%		Ref	–		Ref	–
Yes	493	41.0%		1.460	1.192–2.078		1.550	1.153–2.165
Surgery of the primary site			<0.001			<0.001		
No	768	63.8%		Ref	–		Ref	–
Yes	435	36.2%		1.682	1.369–2.250		1.719	1.288–2.630
Antiviral therapy			<0.001			0.346		
No	420	34.9%		Ref				
Yes	783	65.1%		1.344	1.134–1.735			
Drug usage			<0.001			<0.001		
No‐use	/	/		Ref	–		Ref	–
Only TKI	/	/		1.233	1.089–1.492		1.197	1.064–1.364
Only ICI	/	/		1.388	1.169–1.855		1.402	1.088–1.912
TKI+ICI	/	/		1.513	1.355–2.046		1.628	1.316–2.267
Lung metastasis			<0.001			<0.001		
No	1084	90.1%		Ref	–		Ref	–
Yes	119	9.9%		2.122	1.581–2.862		1.497	1.170–2.113
Comorbidities								
Diabetes mellitus			0.501					
No	1033	85.9%		Ref	–			
Yes	170	14.1%		1.101	0.834–1.473			
Hypertension			0.874					
No	975	81.0%		Ref	–			
Yes	228	19.0%		0.975	0.712–1.336			
Cardiovascular disease			0.371					
No	1099	91.4%		Ref	–			
Yes	104	8.6%		1.211	0.793–1.849			
Chronic kidney disease			0.310					
No	1150	95.6%		Ref	–			
Yes	53	4.4%		1.309	0.781–2.196			
COPD			0.692					
No	1171	97.3%		Ref	–			
Yes	32	2.7%		1.118	0.644–1.940			

*Abbreviations*: AFP: alpha‐fetoprotein; ALBI: albumin–bilirubin grade; ALP: alkaline phosphatase; ALT: alanine aminotransferase; AST: aspartate aminotransferase; CI: confidence interval; COPD: chronic obstructive pulmonary disease; CSPH: clinical significant portal hypertension; GGT: γ‐glutamyl transpeptidase; HBsAg: hepatitis B surface antigen; HCC: hepatocellular carcinoma; HR: hazard ratio; ICI: immune checkpoint inhibitor; PVTT: portal vein tumor thrombosis; RFA: radiofrequency ablation; TKI: tyrosine kinase inhibitor.

### 2.4 Impact of Primary Liver Lesion Resection on Patients With Pulmonary Metastasis

There were a total of 119 patients with initial LM in HCC, among whom 49 received in primary radical treatment (23 underwent hepatectomy/26 received RFA). In the treatment group, the proportion of PVTT was lower, with only four patients (8.2%), while other variables were balanced and showed no statistical differences. However, to ensure comparability between the two groups, 1:1 PSM was conducted. After PSM, all variables between the two groups were balanced, with no statistical differences (Table 3).

**TABLE 3:** Baseline characteristics of hepatocellular carcinoma patients with lung metastasis (LM) underwent RFA/hepatectomy or did not before PSM and after PSM (*n* = 119).

	Before‐PSM			After‐PSM		
	No‐RFA/hepatectomy	RFA/hepatectomy	*p* Value^a^	No‐RFA/hepatectomy	RFA/hepatectomy	*p* Value^a^
*N*	70	49		49	49	
Gender (%)			0.344			0.749
Male	65 (92.9)	43 (87.8)		44 (89.8)	43 (87.8)	
Female	5 (7.1)	6 (12.2)		5 (10.2)	6 (12.2)	
Age (%)			0.200			0.833
<60 y	34 (48.6)	18 (36.7)		17 (34.7)	18 (36.7)	
≥60 y	36 (51.4)	31 (63.3)		32 (65.3)	31 (63.3)	
Tumor max length (%)			0.825			0.683
<5 cm	30 (42.9)	22 (44.9)		20 (40.8)	22 (44.9)	
≥5 cm	40 (57.1)	27 (55.1)		29 (59.2)	27 (55.1)	
Tumor number (%)			0.110			0.233
Single	48 (68.6)	40 (81.6)		35 (71.4)	40 (81.6)	
Multiple	22 (31.4)	9 (18.4)		14 (28.6)	9 (18.4)	
AFP (%)			0.557			0.828
<400 ng/mL	18 (25.7)	15 (30.6)		16 (32.7)	15 (30.6)	
≥400 ng/mL	52 (74.3)	34 (69.4)		33 (67.3)	34 (69.4)	
CSPH (%)			0.905			0.661
No	39 (55.7)	28 (57.1)		26 (53.1)	28 (57.1)	
Yes	31 (44.3)	21 (42.9)		23 (46.9)	21 (42.9)	
PVTT (%)			0.003			0.136
No	48 (68.6)	45 (91.8)		40 (81.6)	45 (91.8)	
Yes	22 (31.4)	4 (8.2)		9 (18.4)	4 (8.2)	
HBsAg (%)			0.158			1.000
No	12 (17.1)	4 (8.2)		4 (8.2)	4 (8.2)	
Yes	58 (82.9)	45 (91.8)		45 (91.8)	45 (91.8)	
ALBI grade (%)			0.507			0.843
1	9 (12.9)	7 (14.3)		6 (12.2)	7 (14.3)	
2	45 (64.3)	35 (71.4)		34 (69.4)	35 (71.4)	
3	16 (22.9)	7 (14.3)		9 (18.4)	7 (14.3)	
ALB (%)			0.151			0.347
<35 g/L	29 (41.4)	14 (28.6)		10 (20.4)	14 (28.6)	
≥35 g/L	41 (58.6)	35 (71.4)		39 (79.6)	35 (71.4)	
ALT (%)			0.444			0.655
<100 U/L	53 (75.7)	34 (69.4)		36 (73.5)	34 (69.4)	
≥100 U/L	17 (24.3)	15 (30.6)		13 (26.5)	15 (30.6)	
AST (%)			0.234			0.418
<80 U/L	28 (40.0)	25 (51.0)		21 (42.9)	25 (51.0)	
≥80 U/L	42 (60.0)	24 (49.0)		28 (57.1)	24 (49.0)	
ALP (%)			0.442			0.790
<100 U/L	53 (75.7)	40 (81.6)		41 (83.7)	40 (81.6)	
≥100 U/L	17 (24.3)	9 (18.4)		8 (16.3)	9 (18.4)	
GGT (%)			1.000			0.681
<60 U/L	40 (57.1)	28 (57.1)		30 (61.2)	28 (57.1)	
≥60 U/L	30 (42.9)	21 (42.9)		19 (38.8)	21 (42.9)	
Smoking history (%)			0.958			0.801
No	16 (22.9)	11 (22.4)		12 (24.5)	11 (22.4)	
Yes	54 (77.1)	38 (77.6)		37 (75.5)	38 (77.6)	
Drinking history (%)			0.864			0.821
No	51 (72.9)	35 (71.4)		36 (73.5)	35 (71.4)	
Yes	19 (27.1)	14 (28.6)		13 (26.5)	14 (28.6)	
Primary curative treatment (%)						
RFA	N/A	26 (53.1)		N/A	26 (53.1)	
Hepatectomy	N/A	23 (46.9)		N/A	23 (46.9)	
Drug usage (%)			0.653			0.883
No use	11 (15.7)	4 (8.2)		6 (12.2)	4 (8.2)	
Only TKI	22 (31.4)	18 (36.7)		17 (34.7)	18 (36.7)	
Only ICI	23 (32.9)	16 (32.7)		17 (34.7)	16 (32.7)	
TKI+ICI	14 (48.6)	11 (22.4)		9 (18.4)	11 (22.4)	

The values in parentheses are percentages unless indicated otherwise.

*Abbreviations*: PSM: propensity score matching; LM: lung metastasis; PVTT: portal vein tumor thrombosis; AFP: alpha‐fetoprotein; HCC: hepatocellular carcinoma; HBsAg: hepatitis B surface antigen; ALBI: albumin–bilirubin grade; ALT: alanine aminotransferase; AST: aspartate aminotransferase; ALP: alkaline phosphatase; GGT: γ‐glutamyl transpeptidase; RFA: radiofrequency ablation; TKI: tyrosine kinase inhibitor; ICI: immune checkpoint inhibitor.

^a^ *χ* ^2^ test with Yates’ correction.

**FIGURE 3:**
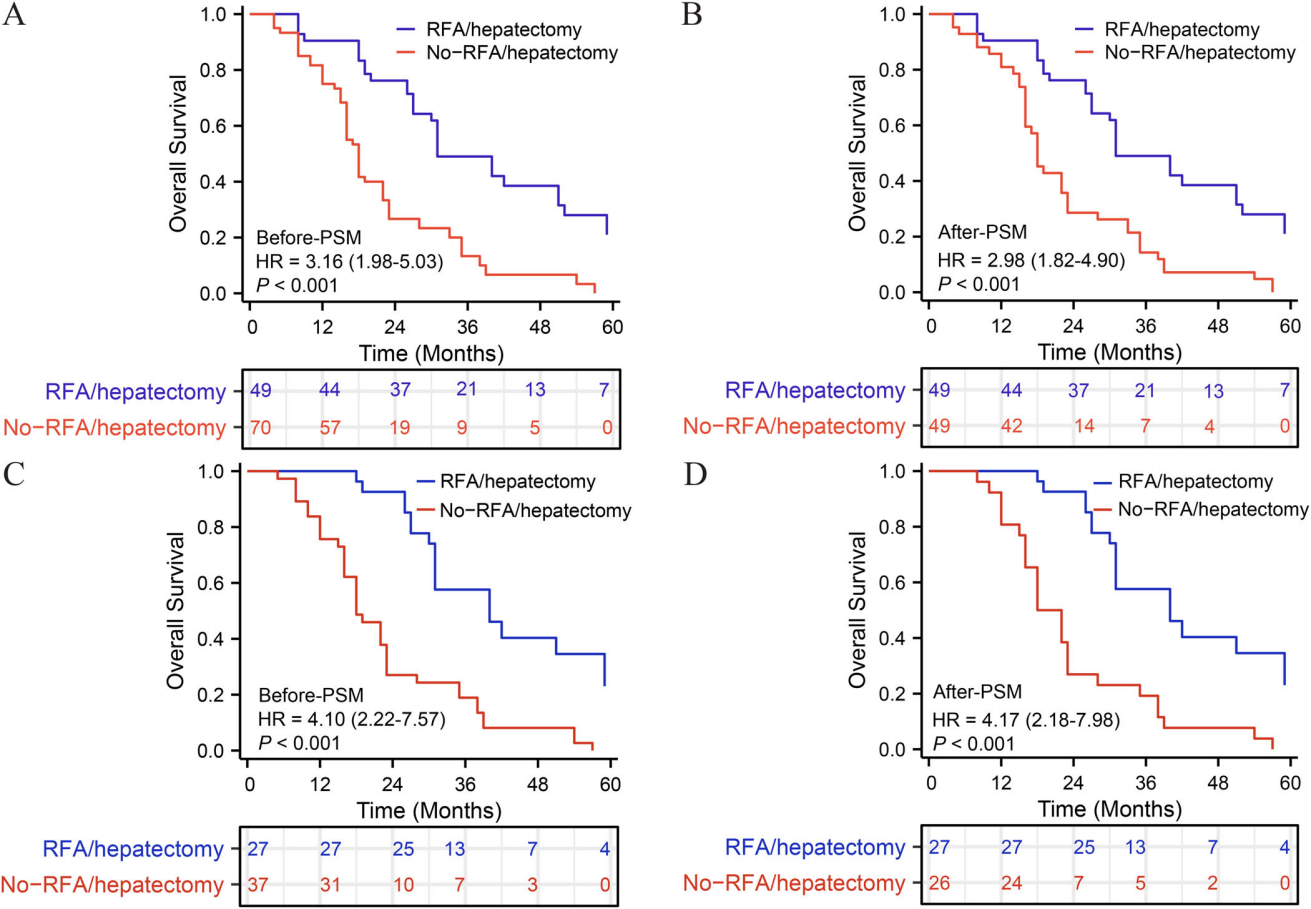
Overall survival rates between patients receiving in primary radiofrequency ablation (RFA)/hepatectomy and those not receiving RFA/hepatectomy in all hepatocellular carcinoma with lung metastasis (HCCLM) patients (A and B) and received immunotherapy HCCLM patients (C and D). (A) OS before propensity score matching. (B) OS after propensity score matching. (C) OS before propensity score matching. (D) OS after propensity score matching.

Before PSM, the 1, 3, and 5‐year overall survival rates in the RFA/hepatectomy group were 89.3, 43.8, and 16.4%, respectively, while in the no‐RFA/hepatectomy group, the rates were 82.1, 21.4, and 0.0%, respectively. Kaplan–Meier curves showed a significant statistical difference in survival rates between the two groups [no‐RFA/hepatectomy vs. RFA/hepatectomy—HR: 3.162 (95% CI: 1.982–5.032; *p* < 0.001)] (Figure 3A). After PSM, Kaplan–Meier curves still showed a significant statistical difference in survival rates between the two groups [no‐RFA/hepatectomy vs. RFA/hepatectomy—HR: 2.983 (95% CI: 1.824–4.903; *p* < 0.001)] (Figure 3B). Among all LM patients who received immunotherapy (only ICI and both ICI/TKI), those who underwent RFA/hepatectomy still had a better prognosis (Figure 3C,D). Immune‐related adverse events associated with PD‐1 therapy were comparable between patients with and without lung metastases, with no significant differences observed across common toxicity categories (Table S8).

### 2.5 The Impact of LM on Radical Treatment of Hepatic in Primary Lesions and Subgroup Analysis

**FIGURE 4:**
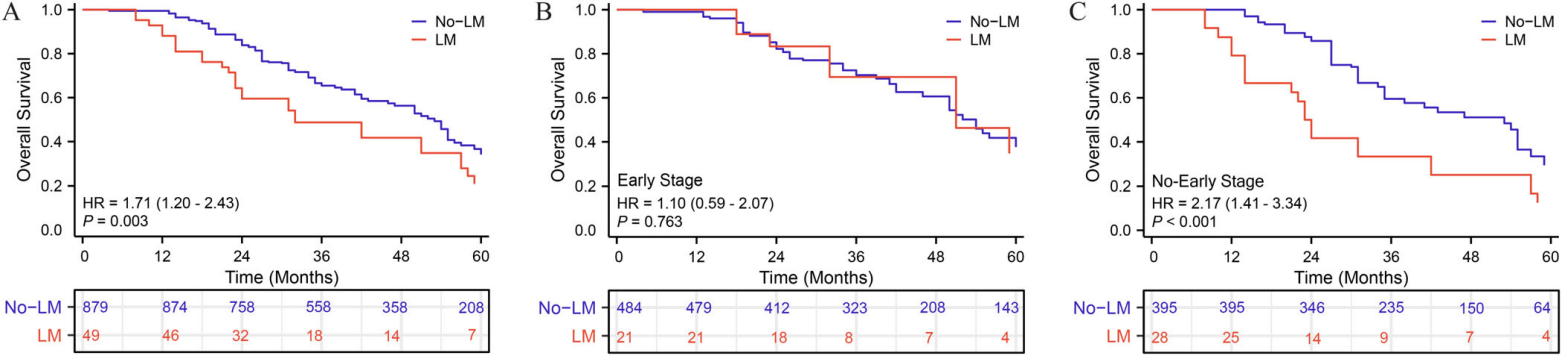
OS rates between patients with LM and those with no‐LM in all patients receiving in primary treatment. (A) OS between two groups. (B) OS between two groups in early stage in primary patients. (C) OS between two groups in no‐early stage in primary patients.

Among the 1203 patients, a total of 928 received locoregional management, while 49 were diagnosed with lung metastases. Baseline characteristics between the two groups are outlined in Table 4. In the LM group, there was a higher proportion of patients with AFP levels ≥ 400 ng/mL, accounting for 69.4%, indicating a statistically significant difference. However, all other variables were balanced between the two groups. Overall, patients in the LM group had a poorer prognosis [LM vs. no‐LM—HR: 1.712 (95% CI: 1.234–2.430; *p* = 0.003)] (Figure 4A). We defined early intrahepatic lesions as tumors located in the liver with a single diameter not exceeding 5 cm or a total number of lesions not exceeding 3, with the largest tumor diameter not exceeding 3 cm and without vascular or lymphatic invasion. This definition aligns with the Milan criteria and corresponds to BCLC stage A for early‐stage HCC. Subgroup analysis revealed comparable prognoses between the LM and no‐LM groups among patients with early intrahepatic lesions [LM vs. no‐LM—HR: 1.102 (95% CI: 0.593–2.071; *p* = 0.763)] (Figure 4B). However, among patients with nonearly intrahepatic lesions, the LM group had a worse prognosis compared with the no‐LM group [LM vs. no‐LM—HR: 2.173 (95% CI: 1.413–3.342; *p* < 0.001)] (Figure 4C). Subgroup forest plots indicated that across different subgroups such as gender, AFP concentration, and the presence of CSPH, the LM group consistently exhibited poorer prognoses. Notably, the presence of lung metastases did not impact prognosis among patients with early intrahepatic lesions (Figure S2).

**TABLE 4:** Baseline characteristics of hepatocellular carcinoma patients with/without lung metastasis (LM) receiving primary curative treatment (RFA and hepatectomy) (*n* = 928).

	Lung metastasis (*n* = 49)	No lung metastasis (*n* = 879)	*p* Value^a^
Gender (%)			0.582
Male	43 (87.8)	746 (84.9)	
Female	6 (12.2)	133 (15.1)	
Age (%)			0.770
<60 y	18 (36.7)	316 (35.9)	
≥60 y	31 (63.3)	563 (64.1)	
Tumor max length (%)			<0.001
<5 cm	22 (44.9)	632 (71.9)	
≥5 cm	27 (55.1)	247 (28.1)	
Tumor number (%)			0.556
Single	40 (81.6)	745 (84.8)	
Multiple	9 (18.4)	134 (15.2)	
AFP (%)			<0.001
<400 ng/mL	15 (30.6)	449 (57.6)	
≥400 ng/mL	34 (69.4)	330 (42.4)	
CSPH (%)			0.069
No	28 (57.1)	611 (69.5)	
Yes	21 (42.9)	268 (30.5)	
HBsAg (%)			0.448
No	4 (8.2)	103 (11.7)	
Yes	45 (91.8)	776 (88.3)	
ALBI grade (%)			0.268
1	7 (14.3)	85 (9.7)	
2	35 (71.4)	711 (80.9)	
3	7 (14.3)	83 (9.4)	
ALB (%)			0.294
<35 g/L	14 (28.6)	316 (35.9)	
≥35 g/L	35 (71.4)	563 (64.1)	
ALT (%)			0.113
<100 U/L	34 (69.4)	694 (79.0)	
≥100 U/L	15 (30.6)	185 (21.0)	
AST (%)			0.322
<80 U/L	25 (51.0)	385 (43.8)	
≥80 U/L	24 (49.0)	494 (56.2)	
ALP (%)			0.040
<100 U/L	40 (81.6)	594 (67.6)	
≥100 U/L	9 (18.4)	285 (32.4)	
GGT (%)			0.191
<60 U/L	28 (57.1)	418 (47.6)	
≥60 U/L	21 (42.9)	461 (52.4)	
Smoking history (%)			0.073
No	11 (22.4)	307 (34.9)	
Yes	38 (77.6)	572 (65.1)	
Drinking history (%)			0.578
No	35 (71.4)	659 (75.0)	
Yes	14 (28.6)	220 (25.0)	
Primary curative treatment (%)			0.983
RFA	26 (53.1)	465 (52.9)	
Hepatectomy	23 (46.9)	414 (47.1)	
Drug usage (%)			0.076
No‐use	6 (10.2)	220 (25.0)	
Only TKI	17 (28.8)	229 (26.1)	
Only ICI	16 (27.1)	188 (21.4)	
TKI+ICI	20 (33.9)	242 (27.5)	
Postoperative pathology^b^			
MVI (%)			0.762
No	16 (69.6)	300 (72.5)	
Yes	7 (30.4)	114 (27.5)	
Satellite Foci (%)			0.525
No	19 (82.6)	361 (87.2)	
Yes	4 (17.4)	53 (12.8)	
Edmondson‐Steiner Grade (%)			0.772
I/II	15 (65.2)	282 (68.1)	
III/IV	8 (34.8)	132 (31.9)	

The values in parentheses are percentages unless indicated otherwise.

*Abbreviations*: LM: lung metastasis; RFA: radiofrequency ablation; AFP: alpha‐fetoprotein; CSPH: clinical significant portal hypertension; HCC: hepatocellular carcinoma; HBsAg: hepatitis B surface antigen; ALBI: albumin–bilirubin grade; ALT: alanine aminotransferase; AST: aspartate aminotransferase; ALP: alkaline phosphatase; GGT: γ‐glutamyl transpeptidase; TKI: tyrosine kinase inhibitor; ICI: immune checkpoint inhibitor.

^a^ *χ* ^2^ test with Yates’ correction.

^b^ Indicates all patients undergoing hepatectomy

### 2.6 Response of Primary Liver Lesions to Systemic Therapy in Patients With and Without LM

We further analyzed the response of primary liver lesions to systemic therapy in patients who did not undergo locoregional treatment, stratified by the presence of LM. In the Only TKI group, patients without LM exhibited a higher objective response rate (ORR) (23.8 vs. 13.0%) and a higher disease control rate (DCR) (71.4 vs. 47.8%) than those with LM. Although the difference in response distribution did not reach statistical significance (*p* = 0.119), a clear trend toward poorer outcomes was observed in the LM subgroup (Table S9).

In the only ICI group, this disparity was more pronounced: LM patients had a markedly lower response rate (8.7 vs. 25.6%) and higher progressive disease rate (60.9 vs. 23.1%) compared with no‐LM patients, with a statistically significant difference in response distribution (*p* = 0.010) (Table S10). Interestingly, in the TKI+ICI combination therapy group, the response rates between LM and no‐LM patients were comparable (ORR 31.0 vs. 20.0%; DCR 78.6% vs. 80.0%), and no significant difference was observed (*p* = 0.850) (Table S11). These findings suggest that combination therapy may help overcome the immune suppression associated with lung metastases and improve the therapeutic response of the primary hepatic tumor.

### 2.7 Follow‐Up of Patients with Lung Metastases and Incidence of Other Organ Metastases in both Groups

During the follow‐up of all 1203 patients, subsequent occurrences of metastases to other organs gradually emerged. In the LM group, there was a higher proportion of patients who developed bone and brain metastases, with statistically significant proportions of 8.5 and 2.0%, respectively, compared with the no‐LM group, where the proportions were 3.4 and 0.6%, respectively (Table S12). Among patients in the LM group, those who experienced extracranial metastases had significantly worse prognoses compared with those without subsequent extracranial metastases (HR = 4.302 (2.461–7.523), *p* < 0.001) (Figure S3).

## 3 Discussion

Liver cancer is characterized by high heterogeneity and aggressiveness, making distant metastasis common, particularly to extracranial sites. Among these, the lungs are the most frequent site of metastasis and are associated with significantly poorer survival and reduced quality of life [6, 12, 13, 14]. As such, proactive intervention in selected patients may offer meaningful prognostic benefits. In our multicenter cohort of 1203 patients, 119 (9.9%) presented with LM at diagnosis. PSM analysis confirmed that LM was associated with significantly worse overall survival. We further evaluated the role of locoregional treatments (hepatectomy or RFA) in LM patients and found that those with early‐stage intrahepatic tumors—defined as a solitary lesion ≤5 cm, or ≤3 nodules with the largest ≤3 cm and no vascular or lymphatic invasion—achieved survival outcomes comparable to no‐LM patients when treated curatively. These findings underscore the importance of individualized locoregional intervention even in patients with metastatic disease. Identifying and treating eligible LM patients with early‐stage intrahepatic disease may help shift current paradigms and improve long‐term outcomes.

In recent years, advances in diagnostic techniques have led to an increased detection of liver cancer patients presenting with lung metastases at initial diagnosis [15]. Prior studies have highlighted the poor prognosis associated with LM in HCC. For example, Wu et al. [9] analyzed SEER data (2010–2015) and reported alarmingly low 1‐, 3‐, and 5‐year overall survival rates of 12.8, 4.0, and 1.6%, respectively. Similarly, Chen et al. [16] found a 51.7% 1‐month mortality rate among HCC patients with lung metastases and developed predictive models using machine learning. Our findings are consistent with these reports in confirming the adverse impact of LM on prognosis. However, both short‐ and long‐term survival outcomes in our cohort were more favorable than those in SEER‐based studies. This discrepancy may be attributed to more aggressive treatment strategies commonly adopted in Asian populations, including adjuvant immunotherapy, resection of metastatic lung lesions, and locoregional therapies for the primary tumor [17, 18]. As our study is based on an Asian cohort predominantly affected by hepatitis B virus (HBV)‐related HCC, the generalizability of these results to Western populations—where hepatitis C virus and nonalcoholic fatty liver disease are more prevalent—remains uncertain. Differences in disease etiology, healthcare systems, and access to systemic therapies such as immunotherapy may also influence outcomes. Therefore, validation through international, multiethnic studies is essential to assess the broader applicability of our findings.

In the 2022 BCLC update, all patients with LM are classified as stage C, limiting treatment options primarily to systemic therapies or conservative care. Although previous studies have shown that resection of lung metastases may improve survival [19, 20, 21], this approach applies to only a small subset of patients with limited and resectable lesions. As most LM cases present with multiple nodules, we excluded patients who underwent LM resection and focused instead on the role of locoregional curative treatment (hepatectomy or RFA) for the primary liver tumor in LM patients. Kaplan–Meier analysis revealed significantly better survival among LM patients who received curative treatment for the primary liver lesion. This was further supported by multivariate Cox regression, which confirmed locoregional therapy as an independent prognostic factor. Our findings align with prior studies from Jung et al. [22] and Lee et al. [23], which emphasized the importance of controlling intrahepatic disease even in advanced or metastatic HCC. Importantly, our results challenge the one‐size‐fits‐all classification of all LM patients as BCLC stage C. By identifying a subset of early‐stage intrahepatic tumors that respond to curative treatment despite the presence of metastasis, we propose a refined management paradigm for metastatic HCC. This personalized approach may improve both survival and quality of life. Moreover, even among LM patients receiving standard immunotherapy, the addition of local treatment to the primary lesion offered significant survival benefits.

While previous studies have explored locoregional treatments in HCC patients with LM, they have not clearly defined which subsets benefit most. For example, Hu et al. [24] reported poor outcomes following aggressive intrahepatic therapy for synchronous LM in a single‐center study. However, their findings may be limited by the small number of patients receiving curative treatment (fewer than 10 cases) and the inclusion of noncurative interventions such as transarterial chemoembolization [25]. In our multicenter analysis, although LM generally predicted worse outcomes, subgroup analysis revealed that LM patients with early‐stage intrahepatic tumors (54.4% of the cohort) had survival comparable to no‐LM patients when treated with hepatectomy or RFA. These findings suggest that curative locoregional therapy can be effective in selected LM patients and challenge the current BCLC staging system, which classifies all LM cases as stage C. We propose that future iterations of the BCLC system consider a distinct subgroup of LM patients—those with favorable intrahepatic disease burden (e.g., meeting Milan criteria—as candidates for combined locoregional and systemic therapy. Such a personalized approach could improve treatment precision and survival outcomes. Therefore, we advocate for proactive intervention in early‐stage cases, rather than uniformly categorizing all HCCLM patients under an advanced disease stage.

In our subgroup analysis of patients who did not receive locoregional treatment, we observed that LM negatively affected the response of the primary liver lesion to systemic therapy. Both TKI and ICI monotherapy showed lower objective response and DCRs in LM patients compared with those without metastasis, with the effect being more pronounced in the ICI group. This suggests that the presence of distant metastases may contribute to systemic immune suppression or tumor microenvironmental changes that diminish the efficacy of single‐agent treatments. Interestingly, this disparity was not observed in patients who received combination therapy with TKI and ICI, indicating that dual blockade may partially overcome the immunosuppressive effects associated with metastasis. These findings support the rationale for using combination regimens in advanced HCC, particularly in patients with extrahepatic spread.

In subsequent follow‐up, it was observed that there were more instances of metastasis to other organs within the LM group, including a higher proportion of bone and brain metastases. Furthermore, LM patients experiencing metastasis to other organs exhibited poorer prognosis. Zhao et al. [26] used the SEER database and found that the incidence of lung metastases was similar between patients with and without bone metastases. This finding differs somewhat from our conclusion, as Zhao et al.’s study was retrospective and cross‐sectional, lacking exploration into the sequence of occurrence. However, Guo et al. [27], comprising a multinational team, recruited 1263 patients initially diagnosed with BM from HCC in SEER, and they identified extrahepatic metastases (lung or brain) as risk factors for bone metastases. Therefore, similar attention should be paid to the occurrence of subsequent metastases to other organs in patients with lung metastases, as this may lead to further deterioration of prognosis.

Although our study represents a large multicenter sample study on lung metastases from liver cancer, there are still some limitations to consider. First, all our samples were from the Asian region, lacking validation from European samples. Second, the etiology of HCC differs from that in the Western population, with HBV infection being predominant in Asian regions. In our analysis, HBV infection status and antiviral therapy did not significantly affect overall survival. However, HBV deoxyribonucleic acid levels were not uniformly available across centers and could not be analyzed. Additionally, multicenter international trials comparing treatment outcomes across different ethnic and demographic populations could further elucidate the best treatment approaches for HCC patients with LM. Such studies would not only help refine the BCLC classification system but also offer more tailored treatment guidelines for clinicians globally.

## 4 Conclusion

This multicenter study confirms that LM significantly worsens prognosis in HCC. However, selected patients with early‐stage intrahepatic disease—defined as a solitary lesion ≤5 cm or up to three lesions with the largest ≤3 cm and no vascular or lymphatic invasion—can benefit from curative locoregional therapies such as hepatectomy or RFA. In this subgroup, survival outcomes were comparable to patients without metastasis. Systemic therapies, including TKIs and ICIs, were associated with improved prognosis. Notably, LM may reduce intrahepatic response to monotherapy, but this effect appears mitigated by combination therapy.

These findings support a more individualized treatment approach for HCC patients with LM. Rather than uniformly classifying all such patients as BCLC stage C, clinical decisions should consider intrahepatic tumor burden and potential responsiveness to combined systemic and locoregional strategies.

## 5 Materials and Methods

### 5.1 Patient

We retrospectively analyzed 1203 HCC patients, among whom 119 patients developed initial lung metastases. Data were collected consecutively from eleven centers between January 2019 and January 2022. All patients were strictly selected and excluded based on predefined criteria. Inclusion criteria were as follows: (1) histopathologically confirmed HCC or diagnosed based on imaging (abdominal ultrasound/computed tomography (CT)/magnetic resonance imaging (MRI)) combined with elevated serum AFP levels, in accordance with international guidelines, (2) initial tumor detection, and (3) For patients undergoing hepatectomy, curative resection (R0) was required. Exclusion criteria were: (1) prior antitumor therapy, (2) other types of tumors, (3) incomplete clinical data and follow‐up information, (4) concurrent other types of tumors, and (5) lung metastasectomy performed. All included patients received TKI and/or ICI therapy, based on decisions made jointly by the medical team and patient's family. This study was approved by the ethics committees of all 11 participating centers. Informed consents were obtained from all patients involved.

### 5.2 HCCLM

These 119 patients were diagnosed with lung metastases at the time of initial presentation. Lung metastases were identified primarily through contrast‐enhanced chest CT, which was performed routinely at diagnosis and follow‐up. In selected cases, additional imaging using MRI was employed when there were equivocal findings or to improve soft‐tissue contrast. The radiological diagnostic criteria for pulmonary metastases included the following features:

1. multiple, well‐circumscribed, round or oval nodules located in bilateral lung fields, often peripherally distributed;
2. absence of benign characteristics such as calcification, fat density, cavitation, or air bronchograms;
3. documented increase in size or number of nodules over time;
4. consistency with the known biological behavior of HCC.

Positron emission tomography–CT (PET–CT) was not routinely used across all participating centers due to limited access and was not a standard component of the diagnostic workflow during the study period.

To reduce interpretation bias, all imaging evaluations were conducted independently by two experienced radiologists at each center, both of whom were blinded to patient treatment allocation and survival outcomes. In cases of discrepancy, a third senior radiologist adjudicated the findings to reach consensus. Inter‐reader agreement was not formally quantified, but substantial consistency was observed in practice.

### 5.3 Curative Treatment of Hepatic Lesions and Procedures

We defined radical treatment of liver lesions as the complete eradication of liver tumors in primary using either hepatectomy or RFA. Intraoperative exploration, including intraoperative ultrasound, was performed to detect all tumor nodules, which were subsequently removed. If surgical resection is performed, negative surgical margins were confirmed by postoperative pathology. postoperative imaging studies such as ultrasound (especially contrast‐enhanced ultrasound), enhanced CT, enhanced MRI, and hepatic angiography showed no residual lesions, no evidence of new vascular or biliary invasion, and no evidence of lymph node metastasis.

All center procedures remained consistent to ensure no bias across centers. Prior to surgery, all patients underwent 3D liver reconstruction to analyze tumor location and its anatomical relationship with major vascular structures, with intraoperative ultrasound used to adjust technical approaches during surgery. All teams performed surgical resection or RFA have overcome the learning curve, with surgical resection including open, laparoscopic, and robot‐assisted laparoscopic approaches. RFA methods included percutaneous, open, and laparoscopic approaches, with the type chosen jointly by the medical team, patient, and family. The details of liver lesion resection during surgery were determined based on tumor location. Residual liver volume >40% was simulated through 3D modeling. Functional reserve (indocyanine green retention rate at 15 min ≤ 45%), tumor size, or number were absolute criteria for resectable tumor treatment. CSPH was defined as a hepatic venous pressure gradient (HVPG) ≥10 mmHg, or the presence of varices or ascites, as per clinical guidelines [28].

For technical details of surgical treatment of in primary lesions, hepatic surgeons perform anatomical resection (AR) or non‐AR based on liver functional reserve, tumor location, and tumor size. AR was preferred initially, with nonanatomical liver resection used if AR was not feasible. Preoperative preparation, incision selection, and lesion exposure were similar to patients undergoing anatomical liver resection, whether open or minimally invasive surgery. Intraoperative ultrasound was used as needed to confirm tumor location and determine the course of the hepatic vein [29]. The Pringle maneuver was used to temporarily block hepatic blood flow, with efforts made to limit the number of occlusions to ≤2 times, each lasting no more than 15 min.

The technical aspects of RFA were managed collaboratively by the surgical and radiology departments to ensure optimal targeting and ablation outcomes. Patients underwent RFA treatment under general anesthesia, guided by ultrasound. The surgical electrode is inserted into the liver cancer lesion, and RFA is performed for 10 min, starting at 20 W and gradually increasing to a maximum power of ≤90 W. When the output power reached 90 W, treatment continues for 2–3 min, with a thermal ablation temperature of 90–95°C. Treatment was continued until tumor tissue degenerates and necroses, and impedance reaches its peak, at which point output power decreases automatically. Three consecutive treatments were performed. The ablation range extended 1 cm beyond the tumor margin to ensure thorough and comprehensive tumor tissue ablation. Intraoperative ultrasound was used again for observation after ablation.

Patients undergoing liver lesion treatment receive routine postoperative care including liver and gastric protection, sedation, pain relief, and close monitoring of vital signs and postoperative complications. Anti‐PD‐1 antibodies used includes camrelizumab, toripalimab, sintilimab, and pembrolizumab. TKI therapy used included sorafenib, lenvatinib, or regorafenib. Tumor response was assessed using the modified response evaluation criteria in solid tumors for HCC, based on contrast‐enhanced imaging (CT or MRI). ORR was defined as the proportion of patients achieving complete response (CR) or partial response (PR), and DCR was defined as the proportion achieving CR, PR, or stable disease.

### 5.4 Follow‐Up

Follow‐up is conducted by specialized follow‐up teams at each center, consisting of two follow‐up nurses and one follow‐up physician, with long‐term uninterrupted follow‐up at all institutions. Most patients are observed based on Chinese guidelines and reference to European and American guidelines. Within the first year after discharge, follow‐up appointments are scheduled every 3 months, during which imaging examinations (such as chest CT and abdominal MRI with contrast, and chest CT) are performed. If patients present specific symptoms such as lower back pain, bone scans or PET–CT scans are conducted to determine the presence of bone metastasis. If neurological symptoms such as headaches and vomiting occur, CT or MRI scans of the brain are performed to determine the presence of brain metastasis. Laboratory tests are conducted, including liver function, kidney function, and complete blood cell counts. After 1 year postdischarge, follow‐up appointments are scheduled every 6 months for patients. Follow‐up continues for all registered patients until confirmed death. Overall survival rate is calculated from the initial diagnosis of HCC at patient admission to death or the last follow‐up time. The follow‐up cut‐off date is March 30, 2025.

### 5.5 Data Analysis

For categorical variables, the chi‐square test or Fisher's exact test was used. For a 2×2 contingency table, if the total sample size *n* ≥ 40, and all cells (a, b, c, d) have expected frequencies (*T*) greater than or equal to 5, the chi‐square test was employed. That is, if *n* ≥ 40, and *T* ≥ 5 for all cells, the chi‐square test was used. If *n* ≥ 40 and at least one 1 ≤ *T* < 5, a corrected chi‐square test was utilized. If *n* < 40 or at least one *T* < 1, Fisher's exact probability test was applied.

To mitigate selection and confounding biases, we employed PSM based on logistic regression, encompassing all variables. PSM was executed utilizing a 1:1 greedy matching algorithm (without replacement) with a caliper set at 0.1 times the standard deviation of the linear predictor. Post‐PSM, covariate distributions between groups were harmonized, exhibiting no significant disparities (*p* > 0.05). OS factors pertinent to HCC were evaluated via both univariate and multivariate Cox regression analyses. Likewise, risk factors contributing to LM were examined through univariate and multivariate logistic regression analyses, with inclusion criteria for the multivariate model being variables demonstrating *p* values <0.05 in the univariate analysis.

Statistical analyses were performed using SPSS 26.0 software, with *p* < 0.05 (two‐side) considered statistically significant. Kaplan–Meier curves were generated using R software (version 4.0.3).

## Author Contributions

Drs F. Xia, Q. Chen, C. Li, and H. Liang contributed equally to this work. Drs F. Xia and Z. Huang had full access to all of the data in the study and take responsibility for the integrity of the data and the accuracy of the data analysis.

Study concept and design: F. Xia, Q. Chen, C. Li, and Z. Huang. Acquisition, analysis, or interpretation of data: F. Xia, Q. Chen, C. Li, and Z. Huang. Drafting of the manuscript: F. Xia and Z. Huang. Critical revision of the manuscript for important intellectual content: Z. Huang, BX. Zhang, H. Liang, and J. Yan. Statistical analysis: F. Xia. Obtained funding: BX. Zhang, Q. Chen, and XP. Chen. Administrative, technical, or material support: J. Yan, Q. Zhang, ZY. Huang, Z. Wu, H. Yin, L. Liu, GB. Xia, J. Zheng, H. Gao, and L. Ren. Study supervision: Z. Huang, BX. Zhang, Q. Chen, and XP. Chen

All authors have read and approved the final manuscript.

## Ethics Statement

The authors are accountable for all aspects of the work in ensuring that questions related to the accuracy or integrity of any part of the work are appropriately investigated and resolved. This research was carried out in accordance with the Declaration of Helsinki (as revised in 2013). Written informed consent for data use was obtained from all patients. The study protocol was approved by the Ethics Committee of Tongji Hospital, Tongji Medical College, within Huazhong University of Science and Technology (202435112).

## Conflicts of Interest

The authors declare no conflicts of interest.

## Supporting information




**Figure S1**: Kaplan‐Meier curves comparing overall survival (OS) among HCC patients with lung metastasis (HCC‐LM) who received different systemic therapy strategies: no treatment, only tyrosine kinase inhibitor (TKI), only immune checkpoint inhibitor (ICI), and combination of TKI and ICI. Combination therapy significantly improved prognosis compared to monotherapy or no treatment (P < 0.001).
**Figure S2**: Forest plot of prognosis in different subgroups among all patients receiving in primary treatment.
**Figure S3**: Comparison of OS between patients with and without occurrence of extra‐organ metastasis in HCCLM patients.
**Table S1**: Comparison of baseline characteristics between included and excluded patients
**Table S2**: Number of patients included from each participating center
**Table S3**: Standardized mean differences (SMD) for baseline variables before and after propensity score matching (PSM) between patients with and without lung metastasis.
**Table S4**: Clinical and Radiological Features of Lung Metastasis in HCC Patients (n = 119)
**Table S5**: Univariate and multivariate logistic regression analyses of risk factors associated with hepatocellular carcinoma lung metastasis.
**Table S6**: Variance Inflation Factor (VIF) for variables in propensity score matching (PSM) between patients with and without lung metastasis.
**Table S7**: Univariate and multivariate Cox regression analyses of prognosis factors associated with HCC with lung metastasis
**Table S8**: Comparison of immune‐related adverse events in HCC patients treated with PD‐1 inhibitors, stratified by lung metastasis status
**Table S9**: Response of the primary liver lesion in HCC patients treated with Only TKI, stratified by lung metastasis status.
**Table S10**: Response of the primary liver lesion in HCC patients treated with Only ICI, stratified by lung metastasis status
**Table S11**: Response of the primary liver lesion in HCC patients treated with TKI + ICI, stratified by lung metastasis status
**Table S12**: Subsequent Organ Metastases in Patients with Hepatocellular Carcinoma, with or without Lung Metastasis (LM)*

## Data Availability

The datasets generated and/or analyzed during the current study are not publicly available due to institutional and ethical restrictions but are available from the corresponding author on reasonable request.

## References

[1] Z. J. Brown , D. I. Tsilimigras , S. M. Ruff , et al., “Management of Hepatocellular Carcinoma: A Review,” JAMA Surgery 158, no. 4 (2023): 410–420. 36790767 10.1001/jamasurg.2022.7989

[2] A. Vogel , T. Meyer , G. Sapisochin , R. Salem , and A. Saborowski . Hepatocellular carcinoma. Lancet (London, England) 400, no. 10360 (2022): 1345–1362. 36084663 10.1016/S0140-6736(22)01200-4

[3] F. Xia , Q. Zhang , G. Xia , et al., “A Pathologic Scoring System for Predicting Postoperative Prognosis in Patients With Ruptured Hepatocellular Carcinoma,” Asian Journal of Surgery 47, no. 7 (2024): 3015‐3025. 38326117 10.1016/j.asjsur.2024.01.139

[4] C. Bosi , M. Rimini , A. Casadei‐Gardini , and E. Giorgio , “Understanding the Causes of Recurrent HCC After Liver Resection and Radiofrequency Ablation,” Expert Review of Anticancer Therapy 23, no. 5 (2023): 503–515. 37060290 10.1080/14737140.2023.2203387

[5] H. Wang , J. Huang , W. Zhang , et al., “Prognostic factors in patients with first diagnosis of hepatocellular carcinoma presenting with pulmonary metastasis and construction of a clinical prediction model,” Updates in Surgery 76, no. 1 (2024): 71–85. 37515700 10.1007/s13304-023-01603-7

[6] J. Feng , Y. He , J. Wan , and Z. Chen , “Pulmonary Metastases in Newly Diagnosed Hepatocellular Carcinoma: A Population‐based Retrospective Study,” HPB: the Official Journal of the International Hepato Pancreato Biliary Association 22, no. 9 (2020): 1295–1304. 31892468 10.1016/j.hpb.2019.12.004

[7] M. Reig , A. Forner , J. Rimola , et al., “BCLC Strategy for Prognosis Prediction and Treatment Recommendation: The 2022 Update,” Journal of Hepatology 76, no. 3 (2022): 681–693. 34801630 10.1016/j.jhep.2021.11.018 PMC8866082

[8] G. Shao , Y. Zhi , Z. Fan , W. Qiu , and G. Lv , “Development and Validation of a Diagnostic and Prognostic Model for Lung Metastasis of Hepatocellular Carcinoma: A Study Based on the SEER Database,” Frontiers in Medicine 10 (2023): 1171023. 37538313 10.3389/fmed.2023.1171023 PMC10394832

[9] C. Wu , X. Ren , and Q. Zhang , “Incidence, Risk Factors, and Prognosis in Patients With Primary Hepatocellular Carcinoma and Lung Metastasis: A Population‐based Study,” Cancer Management and Research 11 (2019): 2759–2768. 31040715 10.2147/CMAR.S192896 PMC6459151

[10] Y. Takahashi , N. Ikeda , J. Nakajima , et al., “Prognostic Analysis of Surgical Resection for Pulmonary Metastasis From Hepatocellular Carcinoma,” World Journal of Surgery 40, no. 9 (2016): 2178–2185. 27255943 10.1007/s00268-016-3580-4

[11] C. M. Lam , C. M. Lo , W. K. Yuen , C. L. Liu , and .F. ST , “Prolonged Survival in Selected Patients Following Surgical Resection for Pulmonary Metastasis From Hepatocellular Carcinoma,” The British Journal of Surgery 85, no. 9 (1998): 1198–1200. 9752858 10.1046/j.1365-2168.1998.00846.x

[12] S. Arora , C. Harmath , R. Catania , A. Mandler , K. J. Fowler , and A. A. Borhani . “Hepatocellular Carcinoma: Metastatic Pathways and Extra‐hepatic Findings,” Abdominal Radiology (New York) 46, no. 8 (2021): 3698–3707. 34091729 10.1007/s00261-021-03151-3

[13] Y. Zhao , Y. Jia , J. Wang , et al., “circNOX4 activates an Inflammatory Fibroblast Niche to Promote Tumor Growth and Metastasis in NSCLC via FAP/IL‐6 Axis,” Molecular Cancer 23, no. 1 (2024): 47. 38459511 10.1186/s12943-024-01957-5 PMC10921747

[14] K. Schütte , R. Schinner , M. P. Fabritius , et al., “Impact of Extrahepatic Metastases on Overall Survival in Patients With Advanced Liver Dominant Hepatocellular Carcinoma: A Subanalysis of the SORAMIC Trial,” Liver Cancer 9, no. 6 (2020): 771–786. 33442545 10.1159/000510798 PMC7768116

[15] A. Abbas , S. Medvedev , N. Shores , et al., “Epidemiology of Metastatic Hepatocellular Carcinoma, a Nationwide Perspective,” Digestive Diseases and Sciences 59, no. 11 (2014): 2813–2820. 24903653 10.1007/s10620-014-3229-9

[16] S. Chen , X. Li , Y. Liang , et al., “Short‐term Prognosis for Hepatocellular Carcinoma Patients With Lung Metastasis: A Retrospective Cohort Study Based on the SEER Database,” Medicine 101, no. 45 (2022): e31399. 36397445 10.1097/MD.0000000000031399 PMC9666127

[17] S. Shan and J. Jia , “The Clinical Management of Hepatocellular Carcinoma in China: Progress and Challenges,” Clinical and Molecular Hepatology 29, no. 2 (2023): 339–341. 36924120 10.3350/cmh.2023.0077 PMC10121312

[18] C. H. Zhang , Y. Cheng , S. Zhang , J. Fan , and Q. Gao , “Changing Epidemiology of Hepatocellular Carcinoma in Asia,” Liver International: Official Journal of the International Association for the Study of the Liver 42, no. 9 (2022): 2029–2041. 35319165 10.1111/liv.15251

[19] K. N. Han , Y. T. Kim , J. H. Yoon , et al., “Role of Surgical Resection for Pulmonary Metastasis of Hepatocellular Carcinoma,” Lung Cancer (Amsterdam, Netherlands) 70, no. 3 (2010): 295–300. 20353879 10.1016/j.lungcan.2010.02.014

[20] K. Kitano , T. Murayama , M. Sakamoto , et al., “Outcome and Survival Analysis of Pulmonary Metastasectomy for Hepatocellular Carcinoma,” European Journal of Cardio‐Thoracic Surgery : Official Journal of the European Association for Cardio‐Thoracic Surgery 41, no. 2 (2012): 376–382. 21727012 10.1016/j.ejcts.2011.05.052

[21] Y. S. Yoon , H. K. Kim , J. Kim , et al., “Long‐term Survival and Prognostic Factors After Pulmonary Metastasectomy in Hepatocellular Carcinoma,” Annals of Surgical Oncology 17, no. 10 (2010): 2795–2801. 20517683 10.1245/s10434-010-1073-5

[22] S. M. Jung , J. W. Jang , C. R. You , et al., “Role of Intrahepatic Tumor Control in the Prognosis of Patients With Hepatocellular Carcinoma and Extrahepatic Metastases,” Journal of Gastroenterology and Hepatology 27, no. 4 (2012): 684–689. 21916984 10.1111/j.1440-1746.2011.06917.x

[23] J. I. Lee , J. K. Kim , D. Y. Kim , et al., “Prognosis of Hepatocellular Carcinoma Patients With Extrahepatic Metastasis and the Controllability of Intrahepatic Lesions,” Clinical & Experimental Metastasis 31, no. 4 (2014): 475–482. 24496959 10.1007/s10585-014-9641-x

[24] J. Wu , P. Zhu , Z. Zhang , et al., “A New Tumor‐associated Antigen Prognostic Scoring System for Spontaneous Ruptured Hepatocellular Carcinoma After Partial Hepatectomy,” Cancer Biology & Medicine 15, no. 4 (2018): 415–424. 30766751 10.20892/j.issn.2095-3941.2018.0095 PMC6372911

[25] Z. Hu , P. Huang , Z. Zhou , et al., “Aggressive Intrahepatic Therapies for Synchronous Hepatocellular Carcinoma With Pulmonary Metastasis,” Clinical & Translational Oncology: Official Publication of the Federation of Spanish Oncology Societies and of the National Cancer Institute of Mexico 20, no. 6 (2018): 729–739. 29110217 10.1007/s12094-017-1779-y

[26] C. Hu , J. Yang , Z. Huang , et al., “Diagnostic and Prognostic Nomograms for Bone Metastasis in Hepatocellular Carcinoma,” BMC Cancer 20, no. 1 (2020): 494. 32487048 10.1186/s12885-020-06995-y PMC7268752

[27] X. Guo , Y. Xu , X. Wang , et al., “Advanced Hepatocellular Carcinoma With Bone Metastases: Prevalence, Associated Factors, and Survival Estimation,” Medical Science Monitor: International Medical Journal of Experimental and Clinical Research 25 (2019): 1105–1112. 30739123 10.12659/MSM.913470 PMC6378855

[28] F. Xia , Z. Huang , Q. Zhang , et al., “Clinically Significant Portal Hypertension (CSPH) on Early‐stage HCC Following Hepatectomy: What's the Impact?,” European Journal of Surgical Oncology: The Journal of the European Society of Surgical Oncology and the British Association of Surgical Oncology 49, no. 4 (2023): 771–779. 36372619 10.1016/j.ejso.2022.11.005

[29] K.‐J. Chu , Y. Kawaguchi , and K. Hasegawa , “Current use of intraoperative ultrasound in modern liver surgery,” Oncology and Translational Medicine 9, no. 4 (2023): 168–175.

