# Supplementary material for: Refining Prognosis and Treatment Strategies Beyond the Barcelona Clinic Liver Cancer Stage in Hepatocellular Carcinoma with Lung Metastases: A Multicenter Cohort Study
Source: MedComm (2020). 2025 Aug 16;6(9):e70306. doi: 10.1002/mco2.70306 (PMC12357102; doi:10.1002/mco2.70306)
Supplement: Supplementary file 1 — Figure S1: Kaplan‐Meier curves comparing overall survival (OS) among HCC patients with lung metastasis (HCC‐LM) who received different systemic therapy strategies: no treatment, only tyrosine kinase inhibitor (TKI), only immune checkpoint inhibitor (ICI), and combination of TKI and ICI. Combination therapy significantly improved prognosis compared to monotherapy or no treatment (P < 0.001). Figure S2: Forest plot of prognosis in different subgroups among all patients receiving in primary treatment. Figure S3: Comparison of OS between patients with and without occurrence of extra‐organ metastasis in HCCLM patients. Table S1: Comparison of baseline characteristics between included and excluded patients Table S2: Number of patients included from each participating center Table S3: Standardized mean differences (SMD) for baseline variables before and after propensity score matching (PSM) between patients with and without lung metastasis. Table S4: Clinical and Radiological Features of Lung Metastasis in HCC Patients (n = 119) Table S5: Univariate and multivariate logistic regression analyses of risk factors associated with hepatocellular carcinoma lung metastasis. Table S6: Variance Inflation Factor (VIF) for variables in propensity score matching (PSM) between patients with and without lung metastasis. Table S7: Univariate and multivariate Cox regression analyses of prognosis factors associated with HCC with lung metastasis Table S8: Comparison of immune‐related adverse events in HCC patients treated with PD‐1 inhibitors, stratified by lung metastasis status Table S9: Response of the primary liver lesion in HCC patients treated with Only TKI, stratified by lung metastasis status. Table S10: Response of the primary liver lesion in HCC patients treated with Only ICI, stratified by lung metastasis status Table S11: Response of the primary liver lesion in HCC patients treated with TKI + ICI, stratified by lung metastasis status Table S12: Subsequent Organ Metastases in Pat [file MCO2-6-e70306-s001.docx]

**Title:** Refining Prognosis and Treatment Strategies Beyond the Barcelona Clinic Liver Cancer Stage in Hepatocellular Carcinoma with Lung Metastases: A Multicenter Cohort Study

Feng Xia^1#^, Qian Chen^1,2#^, Chenyang Li^1#^, Huifang Liang^1#^, Qiao Zhang^3^, Zhiyuan Huang^4^, Zhenheng Wu^5^, Huaxuan Yin^6^, Liping Liu^7^, Jun Zheng^8^, Hengyi Gao^9^, Guobing Xia^10^, Li Ren^11^, Wanguang Zhang^1^, Xiaoping Chen^1^, Jing Yan^12*^, Bixiang Zhang^1*^, Huilan Zhang^l3*^, Zhao Huang^1*^

1. Department of Hepatic Surgery, Tongji Hospital, Tongji Medical College of Huazhong University of Science and Technology, Wuhan, Hubei, China
2. Department of Hepatobiliary Surgery, First Affiliated Hospital of Shihezi University, Shihezi, Xinjiang, China
3. Department of Hepatic Surgery, Zhongshan People's Hospital Affiliated to Guangdong Medical University, Zhongshan, Guangdong, China
4. Department of General Surgery, General Hospital of Central Theater Command, Wuhan, Hubei, China.
5. Department of Hepatopancreatobiliary Surgery, The First Affiliated Hospital of Fujian Medical University, Fuzhou, Fujian, China.
6. Department of Hepatic-biliary-pancreatic Surgery, The First People's Hospital of Foshan, Foshan, China.
7. Department of Hepatobiliary Surgery, Shenzhen People's Hospital, Shenzhen, Guangdong, China.
8. Department of Science and Education, Shenzhen Baoan District People's Hospital, Guangdong, China.
9. Department of Hepatobiliary and Pancreatic Surgery, Shenzhen Longhua District People's Hospital, Guangdong, China.
10. Department of Hepatobiliary and Pancreatic Surgery, Huangshi Central Hospital, Hubei Polytechnic University .Huangshi, Hubei, China.
11. Department of Hepatobiliary Surgery, Affiliated Hospital of Qinghai University, Xining, Qinghai, China.
12. Department of Ultrasound in Medicine, The Second Affiliated Hospital of Zhejiang University School of Medicine, Zhejiang, China.
13. Department of Respiratory and Critical Care Medicine, National Health Commission Key Laboratory of Respiratory Diseases, Tongji Hospital, Tongji Medical College, Huazhong University of Science and Technology, 1095 Jiefang Ave, Wuhan 430030, China.

# These authors contributed equally to the article and should be listed as co-first authors.

*These authors are listed as co-corresponding authors.

**Corresponding authors:**

Zhao Huang, MD, Department of Hepatic Surgery, Tongji Hospital, Tongji Medical College of Huazhong University of Science and Technology, 1095, Jiefang Avenue, Wuhan, China. (huangzhao@tjh.tjmu.edu.cn).

Huilan Zhang, MD, Department of Hepatic Surgery, Tongji Hospital, Tongji Medical College of Huazhong University of Science and Technology, 1095, Jiefang Avenue, Wuhan, China. (Huilanz_76@163.com).

Bixiang Zhang, MD, Department of Hepatic Surgery, Tongji Hospital, Tongji Medical College of Huazhong University of Science and Technology, 1095, Jiefang Avenue, Wuhan, China. (bixiangzhang@hust.edu.cn).

Jing Yan, MD, Department of Hepatic Surgery, Tongji Hospital, Tongji Medical College of Huazhong University of Science and Technology, 1095, Jiefang Avenue, Wuhan, China. (2322030@zju.edu.cn).

**Supplementary Figure Legends**

**Figure S1:** Kaplan-Meier curves comparing overall survival (OS) among HCC patients with lung metastasis (HCC-LM) who received different systemic therapy strategies: no treatment, only tyrosine kinase inhibitor (TKI), only immune checkpoint inhibitor (ICI), and combination of TKI and ICI. Combination therapy significantly improved prognosis compared to monotherapy or no treatment (P < 0.001).

**Figure S2:** Forest plot of prognosis in different subgroups among all patients receiving in primary treatment.

**Figure S3:** Comparison of OS between patients with and without occurrence of extra-organ metastasis in HCCLM patients.

Figure S1


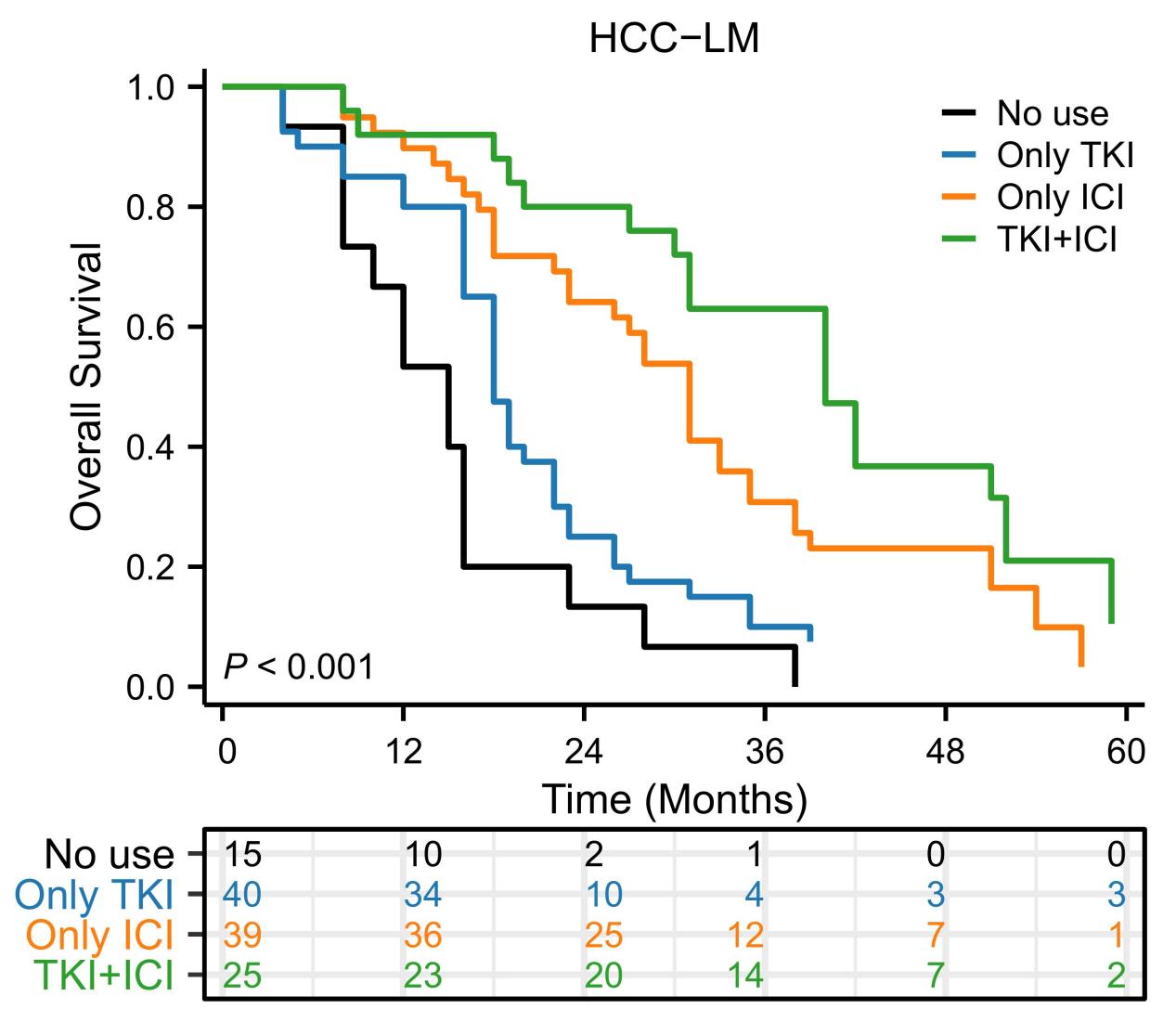


Figure S2


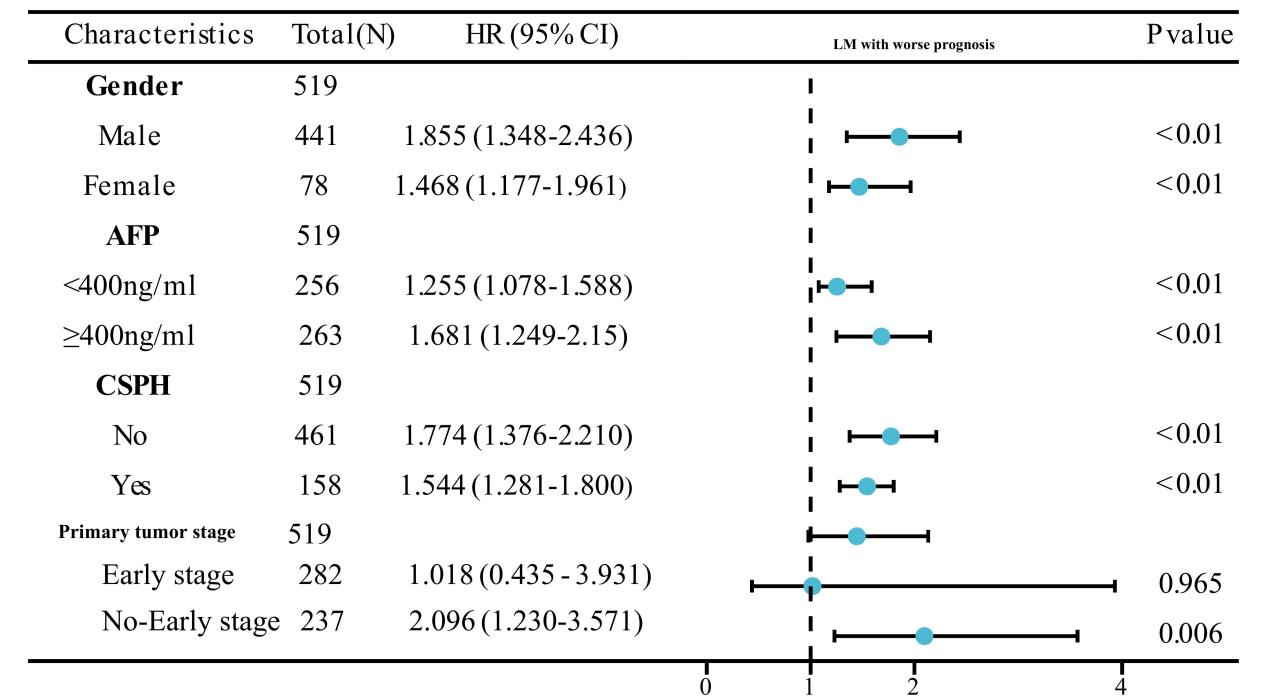


Figure S3


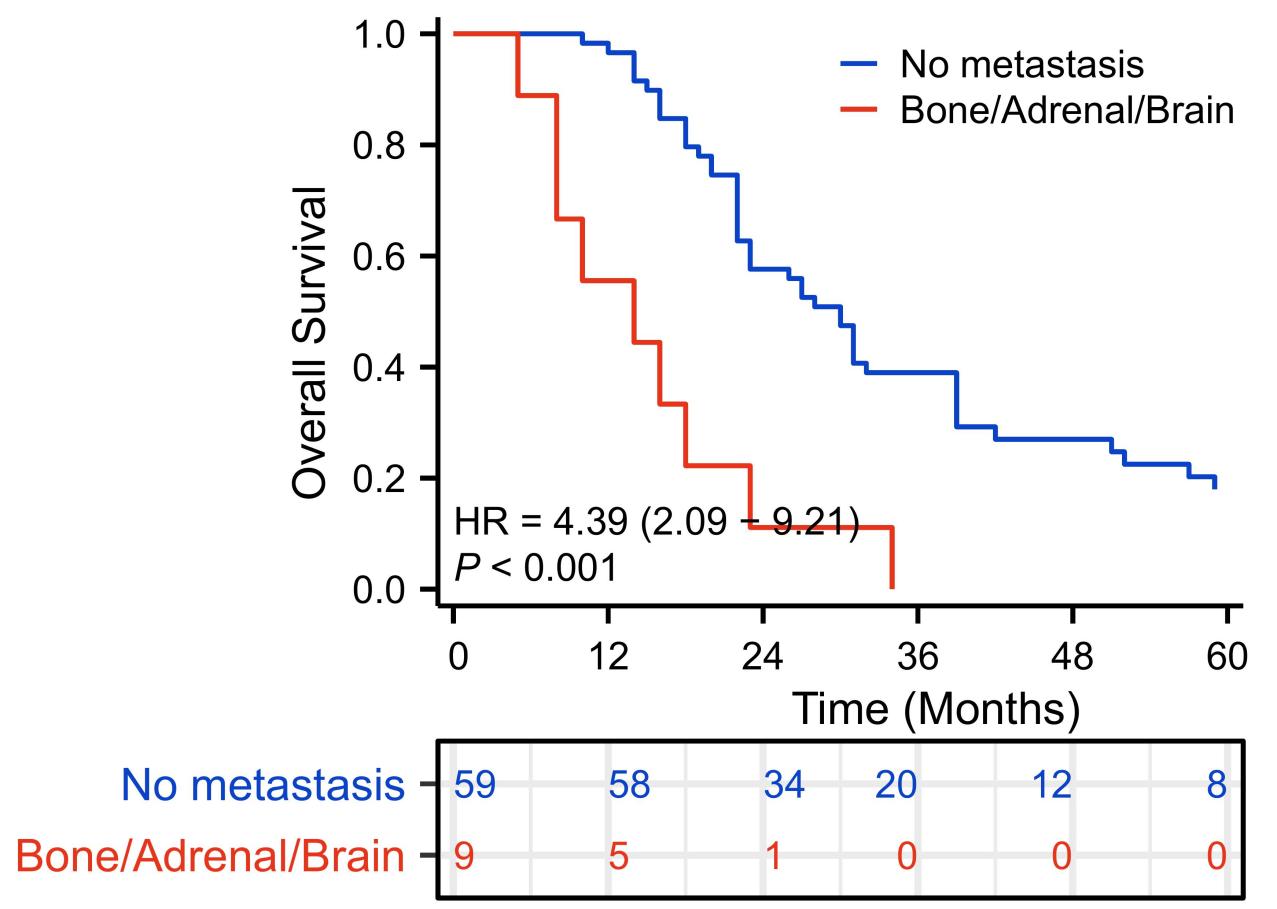


**Table S1.Comparison of baseline characteristics between included and excluded patients**

|  |  | **Included (n=1203)** | **Excluded (n=325)** | **P-value** |
| --- | --- | --- | --- | --- |
| Gender (%) |  |  | | 0.305 |
|  | Male | 1040 (86.5) | 288 (88.6) |  |
|  | Female | 163 (13.5) | 37 (11.4) |  |
| Age (%) |  |  |  | 0.644 |
|  | <60 y | 420 (34.9) | 109 (33.5) |  |
|  | ≥60 y | 783 (65.1) | 216 (66.5) |  |
| Tumor max length (%) | |  |  | 0.307 |
|  | <5cm | 869 (72.2) | 244 (75.1) |  |
|  | ≥5cm | 334 (27.8) | 81 (24.9) |  |
| Tumor number (%) | |  |  | 0.912 |
|  | Single | 905 (75.2) | 253 (75.5) |  |
|  | Multiple | 298 (24.8) | 82 (24.5) |  |
| AFP (%) |  |  |  | 0.375 |
|  | <400ng/ml | 596 (49.5) | 152 (46.8) |  |
|  | ≥400ng/ml | 607 (50.5) | 173 (53.2) |  |
| CSPH (%) |  |  |  | 0.152 |
|  | No | 847 (70.4) | 242 (74.5) |  |
|  | Yes | 356 (29.6) | 83 (25.5) |  |
| PVTT (%) |  |  |  | 0.205 |
|  | No | 1066 (88.6) | 296 (91.1) |  |
|  | Yes | 137 (11.4) | 29 (8.9) |  |
| HBsAg (%) |  |  |  | 0.8731 |
|  | No | 167 (13.9) | 44 (13.5) |  |
|  | Yes | 1036 (86.1) | 281 (86.5) |  |
| ALBI grade (%) |  |  |  | 0.769 |
|  | 1 | 124 (10.3) | 37 (11.4) |  |
|  | 2 | 882 (73.3) | 232 (71.4) |  |
|  | 3 | 197 (16.4) | 56 (17.2) |  |
| ALB (%) |  |  |  | 0.719 |
|  | <35g/L | 379 (31.5) | 99 (30.5) |  |
|  | ≥35g/L | 824 (68.5) | 226 (69.5) |  |
| ALT (%) |  |  |  | 0.409 |
|  | <100U/L | 933 (77.6) | 259 (79.7) |  |
|  | ≥100U/L | 270 (22.4) | 66 (20.3) |  |
| AST (%) |  |  |  | 0.722 |
|  | <80U/L | 627 (52.1) | 173 (53.2) |  |
|  | ≥80U/L | 576 (47.9) | 152 (46.8) |  |
| ALP (%) |  |  |  | 0.364 |
|  | <100U/L | 915 (76.1) | 255 (78.5) |  |
|  | ≥100U/L | 288 (23.9) | 70 (21.5) |  |
| GGT (%) |  |  |  | 0.458 |
|  | <60U/L | 631 (52.5) | 178 (54.8) |  |
|  | ≥60U/L | 572 (47.5) | 147 (45.2) |  |
| Smoking history (%) |  |  |  | 0.104 |
|  | No | 405 (33.7) | 118 (36.3) |  |
|  | ≤5 y | 583 (48.5) | 165 (50.8) |  |
|  | >5 y | 215 (17.9) | 42 (12.9) |  |
| Drinking history (%) |  |  |  | 0.431 |
|  | No | 921 (76.6) | 242 (74.5) |  |
|  | Yes | 282 (23.4) | 83 (25.5) |  |
| Antiviral therapy (%) |  |  |  | 0.236 |
|  | No | 420 (34.9) | 125 (38.5) |  |
|  | Yes | 783 (65.1) | 200 (61.5) |  |
| Comorbidities |  |  |  |  |
| Diabetes mellitus (%) |  | 170 (14.1) | 42 (12.9) | 0.639 |
| Hypertension (%) |  | 228 (19.0) | 68 (20.9) | 0.472 |
| Cardiovascular disease (%) |  | 104 (8.6) | 32 (9.8) | 0.572 |
| Chronic kidney disease (%) |  | 53 (4.4) | 16 (4.9) | 0.804 |
| COPD (%) |  | 32 (2.7) | 7 (2.2) | 0.753 |

Abbreviations: COPD: Chronic obstructive pulmonary disease; PVTT: portal vein tumor thrombosis; AFP: alpha-fetoprotein; HCC: hepatocellular carcinoma; HBsAg: hepatitis B surface antigen; ALBI: albumin–bilirubin grade; ALT: alanine aminotransferase; AST: aspartate aminotransferase; ALP: alkaline phosphatase; GGT: γ-glutamyl transpeptidase.

**Table S2. Number of patients included from each participating center**

| **No.** | **Participating Center** | **Number of Patients** |
| --- | --- | --- |
| 1 | Tongji Hospital of Tongji Medical College of Huazhong University of Science and Technology | 212 |
| 2 | The Second Affiliated Hospital of Zhejiang University School of Medicine | 144 |
| 3 | Zhongshan People's Hospital | 98 |
| 4 | General Hospital of Central Theater Command | 87 |
| 5 | The First Affiliated Hospital of Fujian Medical University | 107 |
| 6 | The First People's Hospital of Foshan | 101 |
| 7 | Shenzhen People's Hospital | 116 |
| 8 | Shenzhen Baoan District People's Hospital | 93 |
| 9 | Shenzhen Longhua District People's Hospital | 79 |
| 10 | Huangshi Central Hospital | 90 |
| 11 | Affiliated Hospital of Qinghai University | 76 |
| Total |  | 1203 |

**Table S3. Standardized mean differences (SMD) for baseline variables before and after propensity score matching (PSM) between patients with and without lung metastasis.**

| **Variable** | **SMD Before Matching** | **SMD After Matching** |
| --- | --- | --- |
| Age ≥60 | 0.201 | 0.084 |
| Sex (Male) | 0.150 | 0.030 |
| Tumor size ≥5cm | 0.592 | 0.084 |
| Tumor number (Multiple) | 0.034 | 0.000 |
| AFP ≥400 ng/ml | 0.494 | 0.000 |
| CSPH (Yes) | 0.327 | 0.000 |
| PVTT (Yes) | 0.327 | 0.022 |
| HBsAg (Yes) | 0.368 | 0.010 |
| ALBI Grade 3 | 0.084 | 0.000 |
| ALB <35g/L | 0.108 | 0.003 |
| ALT ≥100U/L | 0.114 | 0.001 |
| AST ≥80U/L | 0.050 | 0.002 |
| ALP ≥100U/L | 0.057 | 0.001 |
| GGT ≥60U/L | 0.000 | 0.005 |
| Smoking History (Yes) | 0.200 | 0.061 |
| Diabetes mellitus | 0.031 | 0.023 |
| Hypertension | 0.038 | 0.000 |
| Cardiovascular disease | 0.055 | 0.030 |
| Chronic kidney disease | 0.010 | 0.000 |
| COPD | 0.047 | 0.053 |

Abbreviations: AFP, alpha-fetoprotein; ALB, albumin; ALBI, albumin-bilirubin grade; ALP, alkaline phosphatase; ALT, alanine aminotransferase; AST, aspartate aminotransferase; COPD, chronic obstructive pulmonary disease; CSPH, clinically significant portal hypertension; GGT, gamma-glutamyl transpeptidase; HBsAg, hepatitis B surface antigen; PSM, propensity score matching; PVTT, portal vein tumor thrombosis; SMD, standardized mean difference.

**Table S4. Clinical and Radiological Features of Lung Metastasis in HCC Patients (n=119)**

|  |  | **All LM HCC Patients** |
| --- | --- | --- |
| Number of Metastases |  |  |
|  | 1 | 29(24.4) |
|  | > 1 | 90(75.6) |
| Size of Largest Nodule |  |  |
|  | ≤ 2 cm | 96(80.7) |
|  | > 2cm | 23(19.3) |
| Metastasis Progression |  |  |
|  | Stable | 77(64.7) |
|  | Progressive | 42(35.3) |
| Location of Metastases |  |  |
|  | Upper Lung | 36(30.3) |
|  | Lower Lung | 47(39.5) |
|  | Bilateral | 36(30.2) |

Abbreviations: HCC: Hepatocellular Carcinoma; LM: Lung Metastasis

**Table S5. Univariate and multivariate logistic regression analyses of risk factors associated with hepatocellular carcinoma lung metastasis.**

|  | **Survival** | |  | **Univariate Analysis** | | |  | **Multivariate Analysis** | | |
| --- | --- | --- | --- | --- | --- | --- | --- | --- | --- | --- |
| **Variables** | **Number** | **%** |  | **P** | **OR** | **95% CI** |  | **P** | **OR** | **95% CI** |
| Gender |  |  |  | 0.366 |  |  |  |  |  |  |
| Male | 1040 | 86.5% |  |  | 1.215 | 0.805-1.835 |  |  |  |  |
| Female | 163 | 13.5% |  |  | Ref | - |  |  |  |  |
| Age (years) |  |  |  | 0.023 |  |  |  | 0.133 |  |  |
| <60 | 420 | 35.0% |  |  | Ref | - |  |  | Ref | - |
| ≥60 | 783 | 65.0% |  |  | 1.234 | 1.077-1.412 |  |  | 1.164 | 0.813-1.446 |
| Tumor max length(cm) |  |  |  | 0.031 |  |  |  | 0.018 |  |  |
| <5 | 869 | 72.2% |  |  | Ref | - |  |  | Ref | - |
| ≥5 | 334 | 27.8% |  |  | 1.308 | 1.033-1.723 |  |  | 1.455 | 1.108-1.826 |
| Tumor number |  |  |  | <0.001 |  |  |  | <0.001 |  |  |
| Single | 905 | 75.2% |  |  | Ref | - |  |  | Ref | - |
| Multiple | 298 | 24.8% |  |  | 1.851 | 1.279-2.691 |  |  | 1.500 | 1.187-1.942 |
| AFP (ng/ml) |  |  |  | <0.001 |  |  |  | <0.001 |  |  |
| <400 | 596 | 49.5% |  |  | Ref | - |  |  | Ref | - |
| ≥400 | 607 | 50.5% |  |  | 1.208 | 1.132-1.610 |  |  | 1.322 | 1.082-1.916 |
| CSPH |  |  |  | 0.077 |  |  |  | 0.588 |  |  |
| No | 847 | 70.4% |  |  | Ref | - |  |  | Ref | - |
| Yes | 356 | 29.6% |  |  | 1.588 | 1.164-2.307 |  |  | 1.281 | 0.564-2.022 |
| PVTT |  |  |  | 0.001 |  |  |  | <0.001 |  |  |
| No | 1066 | 88.6% |  |  | Ref | - |  |  | Ref | - |
| Yes | 117 | 11.4% |  |  | 1.512 | 1.279-1.912 |  |  | 1.788 | 1.365-2.282 |
| ALBI |  |  |  | 0.772 |  |  |  |  |  |  |
| 1 | 124 | 10.3% |  |  | Ref | - |  |  |  |  |
| 2~3 | 1079 | 89.7% |  |  | 1.061 | 0.597-1.605 |  |  |  |  |
| ALT(U/L) |  |  |  | 0.296 |  |  |  |  |  |  |
| <100 | 933 | 77.6% |  |  | Ref | - |  |  |  |  |
| ≥100 | 270 | 22.4% |  |  | 1.204 | 0.812-1.592 |  |  |  |  |
| AST(U/L) |  |  |  | 0.285 |  |  |  |  |  |  |
| <80 | 627 | 52.1% |  |  | Ref | - |  |  |  |  |
| ≥80 | 576 | 47.9% |  |  | 1.138 | 0.946-1.350 |  |  |  |  |
| ALP(U/L) |  |  |  | <0.001 |  |  |  | 0.155 |  |  |
| <100 | 915 | 76.1% |  |  | Ref | - |  |  | Ref | - |
| ≥100 | 288 | 23.9% |  |  | 1.228 | 1.176-1.851 |  |  | 1.205 | 0.766-1.524 |
| GGT(U/L) |  |  |  | <0.001 |  |  |  | 0.367 |  |  |
| <60 | 631 | 52.5% |  |  | Ref | - |  |  | Ref | - |
| ≥60 | 572 | 47.5% |  |  | 1.364 | 1.095-1.724 |  |  | 1.299 | 0.810-1.566 |
| Drinking history |  |  |  | 0.387 |  |  |  |  |  |  |
| No | 921 | 76.6% |  |  | Ref | - |  |  |  |  |
| Yes | 282 | 23.4% |  |  | 1.086 | 0.702-1.622 |  |  |  |  |
| Smoking history |  |  |  | <0.001 |  |  |  | <0.001 |  |  |
| No | 405 | 33.7% |  |  | Ref | - |  |  | Ref | - |
| ≤5 y | 583 | 48.5% |  |  | 1.351 | 0.698-1.922 |  |  | 1.299 | 0.825-1.731 |
| >5 y | 215 | 17.8% |  |  | 1.920 | 1.388-3.014 |  |  | 2.089 | 1.496-3.281 |

Abbreviations: OR: Odd ratio; CI: confidence interval; AFP: alpha-fetoprotein; CSPH: Clinical Significant Portal Hypertension; HCC: hepatocellular carcinoma; PVTT: Portal Vein Tumor Thrombosis; HBsAg: hepatitis B surface antigen; ALBI: albumin–bilirubin grade; ALT: alanine aminotransferase; AST: aspartate aminotransferase; ALP: alkaline phosphatase; GGT: γ-glutamyl transpeptidase

**Table S6. Variance Inflation Factor (VIF) for variables in propensity score matching (PSM) between patients with and without lung metastasis.**

| **Variable** | **VIF** |
| --- | --- |
| Age | 1.43 |
| Sex | 1.52 |
| HBV status | 1.35 |
| Cirrhosis | 1.61 |
| Child–Pugh class | 1.48 |
| Tumor size ≥5cm | 1.39 |
| Tumor number ≥2 | 1.27 |
| AFP ≥400 ng/ml | 1.44 |
| CSPH | 1.18 |
| PVTT | 1.21 |
| Diabetes mellitus | 1.17 |
| Hypertension | 1.19 |
| Cardiovascular disease | 1.20 |
| Chronic kidney disease | 1.16 |
| COPD | 1.13 |

Abbreviations: AFP: alpha-fetoprotein; COPD: chronic obstructive pulmonary disease; CSPH: clinically significant portal hypertension; HBV: hepatitis B virus; PVTT: portal vein tumor thrombosis; VIF: variance inflation factor.

**Table S7. Univariate and multivariate Cox regression analyses of prognosis factors associated with HCC with lung metastasis.**

|  |  | **Univariate Analysis** | | |  | **Multivariate Analysis** | | |
| --- | --- | --- | --- | --- | --- | --- | --- | --- |
| **Variables** |  | **P** | **HR** | **95% CI** |  | **P** | **HR** | **95% CI** |
| Gender |  | 0.155 |  |  |  |  |  |  |
| Male |  |  | 1.042 | 0.855-1.677 |  |  |  |  |
| Female |  |  | Ref | - |  |  |  |  |
| Age (years) |  | 0.277 |  |  |  |  |  |  |
| <60 |  |  | Ref | - |  |  |  |  |
| ≥60 |  |  | 1.674 | 0.824-2.133 |  |  |  |  |
| Tumor max length(cm) |  | 0.027 |  |  |  | 0.004 |  |  |
| <5 |  |  | Ref | - |  |  | Ref | - |
| ≥5 |  |  | 1.574 | 1.205-2.577 |  |  | 1.634 | 1.207-2.297 |
| Tumor number |  | <0.001 |  |  |  | <0.001 |  |  |
| Single |  |  | Ref | - |  |  | Ref | - |
| Multiple |  |  | 1.711 | 1.294-2.374 |  |  | 1.664 | 1.305-2.411 |
| AFP (ng/ml) |  | <0.001 |  |  |  | 0.024 |  |  |
| <400 |  |  | Ref | - |  |  | Ref | - |
| ≥400 |  |  | 1.390 | 1.077-1.941 |  |  | 1.421 | 1.211-1.830 |
| CSPH |  | 0.004 |  |  |  | 0.029 |  |  |
| No |  |  | Ref | - |  |  | Ref | - |
| Yes |  |  | 1.688 | 1.290-2.243 |  |  | 1.771 | 1.273-2.342 |
| PVTT |  | 0.002 |  |  |  | <0.001 |  |  |
| No |  |  | Ref | - |  |  | Ref | - |
| Yes |  |  | 2.364 | 1.924-3.157 |  |  | 2.107 | 1.692-2.813 |
| ALBI |  | 0.275 |  |  |  |  |  |  |
| 1 |  |  | Ref | - |  |  |  |  |
| 2~3 |  |  | 1.364 | 0.692-1.834 |  |  |  |  |
| ALB(g/L) |  | 0.377 |  |  |  |  |  |  |
| <35 |  |  | Ref | - |  |  |  |  |
| ≥35 |  |  | 0.865 | 0.657-1.314 |  |  |  |  |
| ALT(U/L) |  | 0.357 |  |  |  |  |  |  |
| <100 |  |  | Ref | - |  |  |  |  |
| ≥100 |  |  | 1.527 | 0.697-2.344 |  |  |  |  |
| AST(U/L) |  | 0.674 |  |  |  |  |  |  |
| <80 |  |  | Ref | - |  |  |  |  |
| ≥80 |  |  | 1.377 | 0.847-1.824 |  |  |  |  |
| ALP(U/L) |  | 0.641 |  |  |  |  |  |  |
| <100 |  |  | Ref | - |  |  |  |  |
| ≥100 |  |  | 1.244 | 0.714-1.825 |  |  |  |  |
| GGT(U/L) |  | 0.478 |  |  |  |  |  |  |
| <60 |  |  | Ref | - |  |  |  |  |
| ≥60 |  |  | 1.285 | 0.864-1.671 |  |  |  |  |
| Drinking history |  | 0.366 |  |  |  |  |  |  |
| No |  |  | Ref | - |  |  |  |  |
| Yes |  |  | 1.527 | 0.814-2.043 |  |  |  |  |
| Smoking history |  | 0.724 |  |  |  |  |  |  |
| No |  |  | Ref | - |  |  |  |  |
| Yes |  |  | 1.492 | 0.831-1.825 |  |  |  |  |
| RFA of the primary site |  | <0.001 |  |  |  | <0.001 |  |  |
| No |  |  | Ref | - |  |  | Ref | - |
| Yes |  |  | 1.356 | 1.177-1.853 |  |  | 1.422 | 1.175-2.014 |
| Surgery of the primary site |  | <0.001 |  |  |  | <0.001 |  |  |
| No |  |  | Ref | - |  |  | Ref | - |
| Yes |  |  | 1.588 | 1.291-2.133 |  |  | 1.571 | 1.144-2.320 |
| Antiviral therapy |  | <0.001 |  |  |  | 0.177 |  |  |
| No |  |  | Ref |  |  |  | Ref | - |
| Yes |  |  | 1.428 | 1.114-1.835 |  |  | 1.314 | 0.867-1.916 |
| Drug usage |  | <0.001 |  |  |  | <0.001 |  |  |
| No-use |  |  | Ref | - |  |  | Ref | - |
| Only TKI |  |  | 1.177 | 1.067-1.521 |  |  | 1.128 | 1.055-1.413 |
| Only ICI |  |  | 1.299 | 1.173-1.682 |  |  | 1.341 | 1.125-1.824 |
| TKI+ICI |  |  | 1.466 | 1.247-1.833 |  |  | 1.564 | 1.296-2.043 |
| Features of Lung Metastasis |  |  |  |  |  |  |  |  |
| Number of Metastases |  | <0.001 |  |  |  | 0.006 |  |  |
| No |  |  | Ref | - |  |  | Ref | - |
| Yes |  |  | 1.264 | 1.122-1.832 |  |  | 1.251 | 1.134-1.725 |
| Size of Largest Nodule |  | <0.001 |  |  |  | 0.010 |  |  |
| ≤ 2 cm |  |  | Ref | - |  |  | Ref | - |
| > 2cm |  |  | 1.377 | 1.196-1.712 |  |  | 1.427 | 1.213-1.829 |
| Metastasis Progression |  | <0.001 |  |  |  | 0.002 |  |  |
| Stable |  |  | Ref | - |  |  | Ref | - |
| Progressive |  |  | 1.402 | 1.200-1.816 |  |  | 1.394 | 1.155-1.717 |
| Location of Metastases |  | 0.442 |  |  |  |  |  |  |
| Upper Lung |  |  | Ref | - |  |  |  |  |
| Lower Lung |  |  | 1.142 | 0.816-1.336 |  |  |  |  |
| Bilateral |  |  | 1.058 | 0.769-1.432 |  |  |  |  |

Abbreviations: HR: Hazard ratio; CI: confidence interval; AFP: alpha-fetoprotein; CSPH: Clinical Significant Portal Hypertension; HCC: hepatocellular carcinoma; PVTT: Portal Vein Tumor Thrombosis; HBsAg: hepatitis B surface antigen; ALBI: albumin–bilirubin grade; ALT: alanine aminotransferase; AST: aspartate aminotransferase; ALP: alkaline phosphatase; GGT: γ-glutamyl transpeptidase; RFA: Radiofrequency ablation; TKI: Tyrosine Kinase Inhibitor; ICI: Immune Checkpoint Inhibitor

**Table S8. Comparison of immune-related adverse events in HCC patients treated with PD-1 inhibitors, stratified by lung metastasis status**

| **Adverse Event** | **LM Group (n = 64)** | **Non-LM Group (n = 511)** | **P-value** |
| --- | --- | --- | --- |
| Fatigue | 12 (18.8%) | 89 (17.4%) | 0.928 |
| Rash | 8 (12.5%) | 75 (14.7%) | 0.781 |
| Pruritus | 6 (9.4%) | 50 (9.8%) | 1.000 |
| Immune-related hepatitis | 5 (7.8%) | 36 (7.0%) | 0.797 |
| Hypothyroidism | 7 (10.9%) | 63 (12.3%) | 0.906 |
| Hyperthyroidism | 2 (3.1%) | 15 (2.9%) | 1.000 |
| Pneumonitis | 2 (3.1%) | 14 (2.7%) | 0.696 |
| Diarrhea | 3 (4.7%) | 22 (4.3%) | 0.751 |
| Elevated ALT | 4 (6.3%) | 30 (5.9%) | 0.783 |
| Elevated AST | 4 (6.3%) | 28 (5.5%) | 0.772 |

Abbreviations: ALT, alanine aminotransferase; AST, aspartate aminotransferase; HCC, hepatocellular carcinoma; LM, lung metastasis; PD-1, programmed cell death protein 1.

**Table S9. Response of the primary liver lesion in HCC patients treated with Only TKI, stratified by lung metastasis status.**

| **Response of primary lesion** | **No-LM (n = 63)** | **LM (n = 23)** |
| --- | --- | --- |
| Partial Response (PR) | 15 (23.8%) | 3 (13.0%) |
| Stable Disease (SD) | 30 (47.6%) | 8 (34.8%) |
| Progressive Disease (PD) | 18 (28.6%) | 12 (52.2%) |
| Objective Response Rate (PR%) | 23.8% | 13.0% |
| Disease Control Rate (PR + SD%) | 71.4% | 47.8% |
| P-value (PR/SD/PD distribution) | 0.119 |  |

Abbreviations: DCR, disease control rate; LM, lung metastasis; No-LM, no lung metastasis; ORR, objective response rate; PD, progressive disease; PR, partial response; SD, stable disease; TKI, tyrosine kinase inhibitor.

**Table S10. Response of the primary liver lesion in HCC patients treated with Only ICI, stratified by lung metastasis status**

| **Response of primary lesion** | **No-LM (n = 39)** | **LM (n = 23)** |
| --- | --- | --- |
| Partial Response (PR) | 10 (25.6%) | 2 (8.7%) |
| Stable Disease (SD) | 20 (51.3%) | 7 (30.4%) |
| Progressive Disease (PD) | 9 (23.1%) | 14 (60.9%) |
| Objective Response Rate (PR%) | 25.6% | 8.7% |
| Disease Control Rate (PR + SD%) | 76.9% | 39.1% |
| P-value (PR/SD/PD distribution) | 0.010 |  |

Abbreviations: DCR, disease control rate; ICI, immune checkpoint inhibitor; LM, lung metastasis; No-LM, no lung metastasis; ORR, objective response rate; PD, progressive disease; PR, partial response; SD, stable disease

**Table S11. Response of the primary liver lesion in HCC patients treated with TKI + ICI, stratified by lung metastasis status**

| **Response of primary lesion** | **No-LM (n = 42)** | **LM (n = 5)** |
| --- | --- | --- |
| Partial Response (PR) | 13 (31.0%) | 1 (20.0%) |
| Stable Disease (SD) | 20 (47.6%) | 3 (60.0%) |
| Progressive Disease (PD) | 9 (21.4%) | 1 (20.0%) |
| Objective Response Rate (PR%) | 31.0% | 20.0% |
| Disease Control Rate (PR + SD%) | 78.6% | 80.0% |
| P-value (PR/SD/PD distribution) | 0.850 |  |

Abbreviations: DCR, disease control rate; ICI, immune checkpoint inhibitor; LM, lung metastasis; No-LM, no lung metastasis; ORR, objective response rate; PD, progressive disease; PR, partial response; SD, stable disease; TKI, tyrosine kinase inhibitor.

**Table S12. Subsequent Organ Metastases in Patients with Hepatocellular Carcinoma, with or without Lung Metastasis (LM)***

|  |  | **No-LM(n=1084)** | **LM(n=119)** | **P-value**^‡^ |
| --- | --- | --- | --- | --- |
| Bone metastasis |  |  | | <0.001 |
|  | No | 1062(98.0%) | 107(91.5%) |  |
|  | Yes | 22(2.0%) | 10(8.5%) |  |
| Adrenal metastasis | |  |  | 0.054 |
|  | No | 1080(99.6%) | 117(98.3%) |  |
|  | Yes | 4(0.4%) | 2 (1.7%) |  |
| Brain metastasis | |  |  | 0.001 |
|  | No | 1078(99.4%) | 114(96.6%) |  |
|  | Yes | 6(0.6%) | 4(3.4%) |  |

The values in parentheses are percentages unless indicated otherwise.

* Other organ metastases occurring during follow-up, regardless of whether corresponding treatment has been received.

^‡^ χ^2^ test with Yates’ correction.

Abbreviations: LM: Lung Metastasis
